# Phylogenomic methods outperform traditional multi-locus approaches in resolving deep evolutionary history: a case study of formicine ants

**DOI:** 10.1186/s12862-015-0552-5

**Published:** 2015-12-04

**Authors:** Bonnie B. Blaimer, Seán G. Brady, Ted R. Schultz, Michael W. Lloyd, Brian L. Fisher, Philip S. Ward

**Affiliations:** Department of Entomology, National Museum of Natural History, Smithsonian Institution, Washington, DC 20560 USA; Department of Entomology, California Academy of Sciences, San Francisco, CA 94118 USA; Department of Entomology and Nematology, University of California-Davis, Davis, CA 95616 USA

**Keywords:** Ultraconserved elements, Insect phylogenomics, Ancient rapid radiations, Formicinae, Ant evolution

## Abstract

**Background:**

Ultraconserved elements (UCEs) have been successfully used in phylogenomics for a variety of taxa, but their power in phylogenetic inference has yet to be extensively compared with that of traditional Sanger sequencing data sets. Moreover, UCE data on invertebrates, including insects, are sparse. We compared the phylogenetic informativeness of 959 UCE loci with a multi-locus data set of ten nuclear markers obtained via Sanger sequencing, testing the ability of these two types of data to resolve and date the evolutionary history of the second most species-rich subfamily of ants in the world, the Formicinae.

**Results:**

Phylogenetic analyses show that UCEs are superior in resolving ancient and shallow relationships in formicine ants, demonstrated by increased node support and a more resolved phylogeny. Phylogenetic informativeness metrics indicate a twofold improvement relative to the 10-gene data matrix generated from the identical set of taxa. We were able to significantly improve formicine classification based on our comprehensive UCE phylogeny. Our divergence age estimations, using both UCE and Sanger data, indicate that crown-group Formicinae are older (104–117 Ma) than previously suggested. Biogeographic analyses infer that the diversification of the subfamily has occurred on all continents with no particular hub of cladogenesis.

**Conclusions:**

We found UCEs to be far superior to the multi-locus data set in estimating formicine relationships. The early history of the clade remains uncertain due to ancient rapid divergence events that are unresolvable even with our genomic-scale data, although this might be largely an effect of several problematic taxa subtended by long branches. Our comparison of divergence ages from both Sanger and UCE data demonstrates the effectiveness of UCEs for dating analyses. This comparative study highlights both the promise and limitations of UCEs for insect phylogenomics, and will prove useful to the growing number of evolutionary biologists considering the transition from Sanger to next-generation sequencing approaches.

**Electronic supplementary material:**

The online version of this article (doi:10.1186/s12862-015-0552-5) contains supplementary material, which is available to authorized users.

## Background

Current target-enrichment and next-generation sequencing techniques allow for the rapid generation of hundreds of loci for use as phylogenetic markers. This is demonstrated by an increasing number of studies, largely conducted on vertebrates (e.g., [1–4]). One of the most promising approaches focuses on capturing ultraconserved elements (UCEs)—regions in the genome that have remained highly conserved across great evolutionary distances. Core UCEs are sequenced together with their more variable flanking regions, producing markers for phylogenetic reconstruction [5, 6]. Recently, this method has been adapted and applied to insects, informing family-level relationships among Hymenoptera (bees, ants and wasps) [7]. Although these prior studies report the successful use of UCEs in phylogenetics, we are not aware of any study directly comparing this phylogenomic method to the longstanding use of multi-locus sequence data in phylogenetics. In an important recent exercise, Gilbert et al. [8] calculated and compared the phylogenetic informativeness of UCEs and several single-copy nuclear markers extracted in silico from eight published fish genomes. Here we address an unresolved phylogenetic problem by simultaneously generating both UCE and traditional Sanger-sequenced data for the same 82 ant species, estimating and directly comparing phylogenies separately produced by each source of information. Many biologists require such an applied comparison as they evaluate the costs and benefits of next-generation techniques over Sanger sequencing in advance of the data collection phases of their next projects.

The evolutionary history and ecological success of the ants (family Formicidae) have been illuminated recently in multiple studies using a variety of approaches. For example, recent molecular phylogenetic research has clarified relationships among and within subfamilies [9–14], while other research has focused on diversification patterns [15–17] or the evolution of successful behaviors [18, 19]. The ant subfamily Formicinae is the second most species-rich subfamily of ants with around 3000 described species, trumped in diversity only by the Myrmicinae [20]. The group contains the well-known, economically important carpenter ants of the genus *Camponotus*, presently the most diverse genus-level clade of ants in the world with over 1,000 described species. Other prominent members of this group include the silk-spinning weaver ants (*Oecophylla*) and spiny ants (*Polyrhachis*), which dominate the forest canopies of the Old World, as well as the yellow crazy ant *Anoplolepis gracilipes*, one of the world’s most destructive and invasive ant species. Despite being stingless, formicines have derived a defensive venom exceptional among the arthropods, formic acid (well described e.g. in *Camponotus*, *Formica*, *Lasius* [21]), and also have been identified recently as the only known dietary source of pumiliotoxins sequestered by dendrobatid poison dart frogs [22]. Many formicine ants also exhibit intriguing slave-making behavior (e.g. *Polyergus, Rossomyrmex*) or other forms of social parasitism (e.g. *Lasius, Plagiolepis*) [23].

Recent phylogenetic research has focused on resolving generic relationships within subfamily-level groups of ants such as the Myrmicinae [14], Ponerinae [13], and Dorylinae [12]. The evolution of the subfamily Formicinae, however, has not yet been comprehensively scrutinized, with the exception of one particular subgroup, the *Prenolepis* genus-group [24, 25]. Prior studies of generic relationships within these subfamilies were based on data sets composed of multiple nuclear loci generated by traditional Sanger sequencing. Particularly in the cases of the Myrmicinae and the Dorylinae, these methods were not able to provide information adequate for entirely resolving lineage diversification [12, 14].

We compare the efficacy of a UCE-based phylogenomic data set to that of a high-quality nuclear-gene data set for resolving phylogenetic relationships and obtaining divergence estimates within formicine ants. To do so, we assembled a data set of 959 UCE loci by means of target enrichment and multiplexed sequencing for 82 formicine taxa, and simultaneously generated a data set of ten PCR-amplified and Sanger-sequenced nuclear loci (eight of these protein-coding) for the same 82 taxa. We then use these combined results to investigate (i) the power of each data set for resolving the phylogeny of the subfamily Formicinae and (ii) the evolutionary and biogeographic history of the subfamily.

## Methods

### Molecular data collection

#### Taxon sampling

A more extensive description of all methods can be found in Additional file 1. Our data set comprised 82 ant species, which represent 48 of the 51 currently-valid formicine genera. We further included eight outgroup taxa from seven other ant subfamilies (Myrmicinae, Ectatomminae, Heteroponerinae, Pseudomyrmecinae, Myrmeciinae, Aneuretinae, Dolichoderinae) belonging to the formicoid clade of ants (sensu Brady et al. [9]), and trees were rooted using the four subfamilies most distantly related to the formicines. Ants for this study were collected at the following locations, and with respective institutions providing authorizations for the capture, collection and exportation: AUSTRALIA: Environmental Protection Agency, Queensland Parks and Wildlife Service; BRUNEI: Universiti Brunei Darussalam and the Brunei Museums; CENTRAL AFRICAN REPUBLIC: Ministère de l'Environnement des Eaux, Forest, Chasses et Pêche; COSTA RICA: Ministerio del Ambiente y Energia; Direction General de Vida Silvestre, Ministerio de Recursos Naturales Energia y Minas; FIJI: Ministry of Fisheries and Forests, Department of Forestry; GABON: National Center for Scientific and Technological Research; HONG KONG: Agriculture, Fisheries and Conservation Department, Kowloon; MADAGASCAR: Ministère de l'Environnement et des Forêts, Madagascar National Parks; MALAYSIA: Sabah Biodiversity Council; UGANDA: Uganda National Council for Science and Technology, Uganda Wildlife Authority; UNITED STATES: National Park Service; and State of California Natural Resources Agency, Department of Parks and Recreation. Vouchers have been deposited at the University of California, Davis, at the National Museum of Natural History, and at the California Academy of Sciences. Additional file 2 lists specimen identifiers; collection data can be found by searching for these CASENT numbers on the AntWeb (www.antweb.org) database. DNA was extracted destructively or non-destructively from worker ants or pupae using a DNeasy Blood and TissueKit (Qiagen, Valencia, CA, USA).

#### Library preparation, target enrichment and sequencing of UCEs

We sheared 2.8–497 ng (139 ng mean) DNA to a target size of approximately 500–600 bp by sonication and used this sheared DNA as input for a modified genomic DNA library preparation protocol following Faircloth et al. ([7], but see Additional file 1). We enriched pooled libraries using a set of 2749 custom-designed probes (MYcroarray, Inc.) targeting 1510 UCE loci in Hymenoptera [7]. We followed library enrichment procedures for the MYcroarray MYBaits kit [26], except we used a 0.1X concentration of the standard MYBaits concentration, and added 0.7 μL of 500 μM custom blocking oligos designed against our custom sequence tags. We used the with-bead approach for PCR recovery of enriched libraries as described in Faircloth et al. [7]. Following post-enrichment PCR, we purified resulting reactions using 1.0X speedbeads and rehydrated the enriched pools in 22 μL EB.

We performed qPCR using a SYBR® FAST qPCR kit (Kapa Biosystems) on a ViiA^TM^ 7 (Life Technologies), and based on the size-adjusted concentrations estimated by qPCR, we pooled libraries at equimolar concentrations and size-selected for 250–800 with a BluePippin (SageScience). The pooled libraries were sequenced using two partial lanes of a 150-bp paired-end Illumina HiSeq 2500 run (U Cornell Genomics Facility). All of the UCE laboratory work was conducted in and with support of the Laboratories of Analytical Biology (L.A.B.) facilities of the National Museum of Natural History (NMNH). Quality-trimmed sequence reads generated as part of this study are available from the NCBI Sequence Read Archive (http://www.ncbi.nlm.nih.gov/sra; SUB1067415).

#### Amplification, Sanger sequencing, and alignment of nuclear loci

Ten nuclear markers commonly used in ant systematics were selected for amplification ([for primers see [9, 11, 27, 28]): Long-wavelength rhodopsin (*LW Rh*, 458 bp), elongation factor 1-alpha F1 (*EF1aF1*, 359 bp), elongation factor 1-alpha F2 (*EF1aF2*, 517 bp), abdominal-A (*abdA*, 606 bp), arginine kinase (*argK*, 673 bp), ultrabithorax (*Ubx*, 630 bp), 18S ribosomal DNA (1851 bp), 28S rDNA (825 bp), wingless (*Wg*, 412 bp) and topoisomerase 1 (*Top1*, 883 bp), for a total of 7214 bp in the aligned data matrix. Amplifications were performed using standard PCR methods outlined in Ward and Downie [27] and cycle sequencing reactions were performed using PCR primers and BigDye ® Terminator ver. 3.1 Cycle Sequencing chemistry. Amplicons were analyzed on ABI 3730 Sequencers © (2011 Life Technologies, Frederick, MA) housed at the College of Biological Sciences DNA Sequencing Facility, University of California, Davis, CA and at L.A.B. at the NMNH, Washington, DC. Sequence data were aligned with MAFFT v7.017 [29]. All newly generated sequences have been deposited in GenBank, under accessions KT443144-KT443783 (see Additional file 2).

### Processing and alignment of UCE data

We trimmed the demultiplexed FASTQ data output for adapter contamination and low-quality bases using Illumiprocessor [30], based on the package Trimmomatic [31]. All further data processing described in the following relied on the PHYLUCE package [6, 32]; a detailed description of this pipeline and its scripts can be found in Additional file 1.

We computed summary statistics on the data and assembled the cleaned reads using Trinity (version trinityrnaseq_r20140717) [33]. To identify contigs representing enriched UCE loci from each species, species-specific contig assemblies were aligned to a FASTA file of all enrichment baits (min_coverage = 50, min_identity = 80), and sequence coverage statistics (avg, min, max) for contigs containing UCE loci were calculated. We created FASTA files for each UCE locus containing sequence data for taxa present at that particular locus and aligned these using MAFFT [29] (min-length = 20, no-trim). We further trimmed our alignments using Gblocks [34]. Initially, we selected the following subsets of UCE alignments depending on the captured UCE loci across taxa: 1) 50 % complete (containing data from ≥ 45 of the 90 taxa for each locus), 2) 60 % complete (≥54 of 90 taxa), 3) 70 % complete (≥63 of 90 taxa) and 4) 95 % (≥85 of 90 taxa).

### Phylogenetic inference

For the 10-gene data set, PartitionFinder v.1.1.1 [35] was used to simultaneously select data partitions and estimate appropriate models of evolution, for subsequent analyses with maximum likelihood (ML) and Bayesian methods. ML analyses were carried out in the programs RAxML v7.7.7 [36] and GARLI v.2.0 [37] and included both best tree and bootstrap searches. Bayesian inference (BI) was performed in MRBAYES 3.2 [38] with 2 independent runs of 40 million generations, summarizing 72000 trees after discarding a burnin of 10 %. MCMC convergence was checked visually and with Bayes Factor comparisons using TRACER v1.6 (http://tree.bio.ed.ac.uk/software/tracer/) and by examining PSRF values in MrBayes .stat output files. All analyses were carried out using parallel processing (one chain per CPU) on a 12-core Intel-processor Apple computer or on the Smithsonian NMNH L.A.B Topaz network of Apple computers with Intel processors.

To select data partitions for the UCE phylogenomic data set, we used a development version of PartitionFinder [39] that depends on the software fast_TIGER (http://dx.doi.org/10.5281/zenodo.12914) and is designed to handle large genome-scale data sets. The UCE data set was analyzed with ML best tree and bootstrap searches (N = 100) in RAxML v8.0.3 [36], initially on a 50 %, 60 %, 70 % and 95 % complete UCE matrix (see above). For subsequent analyses, however, we elected to proceed with the 70 % and 95 % matrices. We also reconstructed gene trees for the 959 UCE loci in the 70 % matrix by performing RAxML analyses (best tree and bootstrap) on individual loci, and used these to construct a subset of UCE data, representing the 100 loci with the best average bootstrap score (UCE-100best hereafter). The four main data sets used for downstream analyses are summarized in Table 1. We calculated phylogenetic informativeness (PI) [40] per nucleotide site for the three UCE and the 10-gene data sets with the software package TAPIR [41] (http://faircloth-lab.github.com/tapir/), a parallelized version of PhyDesign [42].

**Table 1:** Overview of UCE and Sanger data sets

	Sanger - 10-gene	UCE - 70 %	UCE - 100best	UCE - 95 %
Loci	10	959	100	50
Total bp	7214 bp	589757 bp	71611 bp	35619 bp
Mean PI (ingroup)	4.09E-04	7.86E-04	9.39E-04	6.65E-04
Data partitions	12	101	18	18
RAxML	x	x	x	x
GARLI	x	-	-	-
MrBayes	40 Mgen	-	-	-
BEAST	500 Mgen	-	300 Mgen	300 Mgen

Summary of number of loci, length of matrix, mean PI, number of data partitions and type of analyses for the four data sets used in this study. PI = phylogenetic informativeness *sensu* Townsend [40], calculated for ingroup taxa only

We identified five taxa, subtended by long branches, which influenced resolution in analyses of both the UCE and 10-gene data sets. In order to better understand the effects of these taxa on phylogenetic results, we carried out phylogenetic analyses (BI for 10-gene, ML for UCEs) with a series of taxon-reduced data sets. Data matrices as well as the resulting tree files for the four main data sets are deposited in Treebase (http://purl.org/phylo/treebase/phylows/study/TB2:S18146).

### Dating analyses

We inferred divergence dates within the Formicinae from the UCE-100best, UCE-95 %, and the 10-gene data set with the program BEAST v1.8 [43]. We chose these smaller UCE data sets for the dating analysis because BEAST cannot currently handle larger data sets with hundreds of loci such as our full 70 % matrix. We performed analyses on the 10-gene data set with four independent runs and 500 million generations; UCE analyses consisted of two runs of 300 million generations each for 95 % and 100best data sets (see Table 1). All divergence analyses were calibrated by placing calibration priors on nine nodes in the phylogeny (see Additional file 3). Trace files were analyzed in Tracer v1.6 to determine chain convergence and burnin. Tree files were then summarized with LogCombiner v1.8.2 and TreeAnnotator v1.8.2 after discarding a burnin of 20 %. These dating analyses and all phylogenetic analyses on UCEs were performed on the Smithsonian Institution high performance cluster (SI/HPC).

### Biogeographic analyses

We constructed a species distribution matrix to evaluate the biogeographic history of Formicinae (see Additional file 4). We assigned to each terminal taxon the distribution of its species plus that of other species estimated to be more closely related to the terminal taxon than to any other species in our data set. We used the dispersal-extinction-cladogenesis model (DEC, “Lagrange,” [44]) and the statistical DEC model (S-DEC, “Bayes-Lagrange”, [45]) implemented in the program RASP [46] to estimate ancestral ranges from the set of trees and the respective MCC tree from our BEAST analysis on the UCE-100best data set. Under both models, outgroups were removed before the analyses. We followed Ward et al. [14] in designating six biogeographic areas (Neotropical, Nearctic, Palearctic, Afrotropical, Indomalayan and Australasian) and defined different dispersal constraints for two time slices (0–50 Ma and 50–105 Ma) based on paleogeography (Scotese, 2010, PALEOMAP project; http://www.scotese.com/) (see Additional file 5).

## Results

### UCE capture statistics

Multiplexed sequencing of UCEs resulted in an average of 1.6 million reads per sample (see Additional file 6) with an average length of 290 base pairs (bp). An average of 29655 contigs with a mean length of 359.2 bp was assembled by Trinity after adapter- and quality-trimming, with an average coverage of 17.4X. From all of the assembled contigs, we recovered an average of 936 UCE loci per sample with a mean length of 805 bp. The average coverage per captured UCE locus was 92.3X. Following alignment of individual UCE loci, we filtered these data for loci captured for ≥70 % of taxa (UCE-70 %), retaining 959 loci, and for loci captured for ≥95 % of taxa (UCE-95 %), retaining 50 loci. We further selected a data set of 100 loci with the best average bootstrap support for subsequent dating analyses (UCE-100best), because this represented a manageable size for BEAST (whereas analysis of the full 959 loci was not feasible). Concatenation of UCE loci generated matrices of 589757 bp (UCE-70 %), 71611 bp (UCE-100best), and 35619 bp (UCE-95 %). The ten Sanger-sequenced nuclear loci were concatenated into one matrix of 7214 bp of protein-coding and ribosomal DNA data, with no missing data for any taxon. Table 1 provides an overview of these data sets.

### Phylogenetic results

**Fig. 1:**
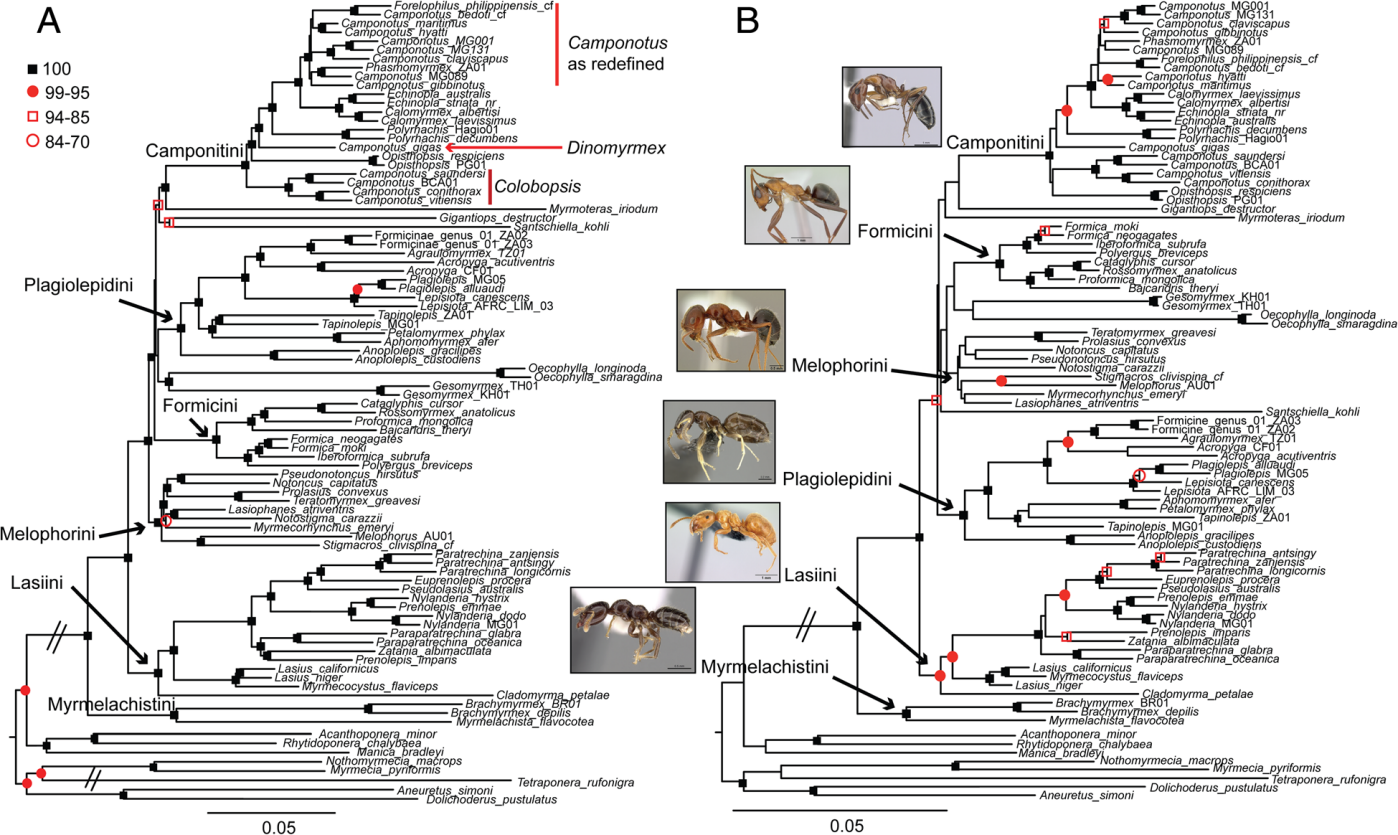
Phylogeny of the subfamily Formicinae. Contrasting phylogenetic trees estimated by **a** the phylogenomic UCE-70 % data set and **b** the "traditional" Sanger-sequencing-generated 10-nuclear-gene data set. Both figures are based on RAxML best tree searches, with RAxML bootstrap values mapped on the respective nodes. The bootstrap searches included 100 and 1152 replicates for UCE and 10-gene data set, respectively. The six larger formicine tribes are indicated. See also Additional file 8

PartitionFinder selected 12 data partitions as the best-fitting scheme for our 10-gene matrix, whereas the UCE-70 %, UCE-95 % and UCE-100best data sets were divided into 101, 18, and 18 partitions, respectively (Table 1). The results of maximum likelihood (ML) best tree and bootstrap searches on the partitioned UCE-70 % data set and 10-gene data sets are summarized in Fig. 1. Analyses of both data sets identified six major, well-supported clades within the Formicinae, outlined below, as well as five isolated genera for which closest relatives remain uncertain. We propose tribal and genus-level revisions to the classification of the subfamily based on our phylogenetic results (as detailed in Additional file 7), intended for formal publication elsewhere (Ward et al., in review).

1. Camponotini: This clade is recovered with high bootstrap support (BS = 100) in both UCE and 10-gene analyses, and includes the genera *Camponotus*, *Polyrhachis*, *Opisthopsis*, *Echinopla*, *Phasmomyrmex*, and *Forelophilus.*
2. Plagiolepidini (redefined): We recovered very good support (BS = 100 in both analyses) for a clade containing the genera *Acropyga, Anoplolepis, Agraulomyrmex, Aphomomyrmex, Lepiosota, Petalomyrmex, Plagiolepis, Tapinolepis*, and an undescribed formicine genus. *Lepisiota* was further recovered as paraphyletic with respect to *Plagiolepis* (Fig. 1).
3. Formicini: All current members of the tribe Formicini form another highly-supported clade in both UCE and 10-gene analyses (BS = 100/100), including *Bajcaridris*, *Cataglyphis*, *Formica, Ibericoformica*, *Rossomyrmex*, *Polyergus*, and *Proformica*.
4. Melophorini (redefined): The UCE phylogeny reveals a well-supported clade (BS = 100) containing *Lasiophanes, Melophorus, Myrmecorhynchus, Notoncus, Pseudonotoncus, Notostigma, Prolasius, Stigmacros*, and *Teratomyrmex*. This clade is also recovered in analyses of the 10-gene data set, but with lower support (BS: GARLI = 64, RAxML = 55; BI/PP: 1.0).
5. Lasiini (redefined): Both UCE and 10-gene data sets further highly support (BS = 100/95) a clade consisting of ten genera: *Cladomyrma, Euprenolepis, Lasius, Myrmecocystus, Nylanderia, Paraparatrechina, Paratrechina, Prenolepis, Pseudolasius* and *Zatania*. Two genera, *Prenolepis* and *Nylanderia*, were further recovered as paraphyletic with respect to each other.
6. Myrmelachistini (resurrected): Both data sets recover *Brachymyrmex* and *Myrmelachista* as sister to all other formicines, forming a highly supported clade (BS = 100/100).

### Performance of UCE versus 10-gene data sets

**Fig. 2:**
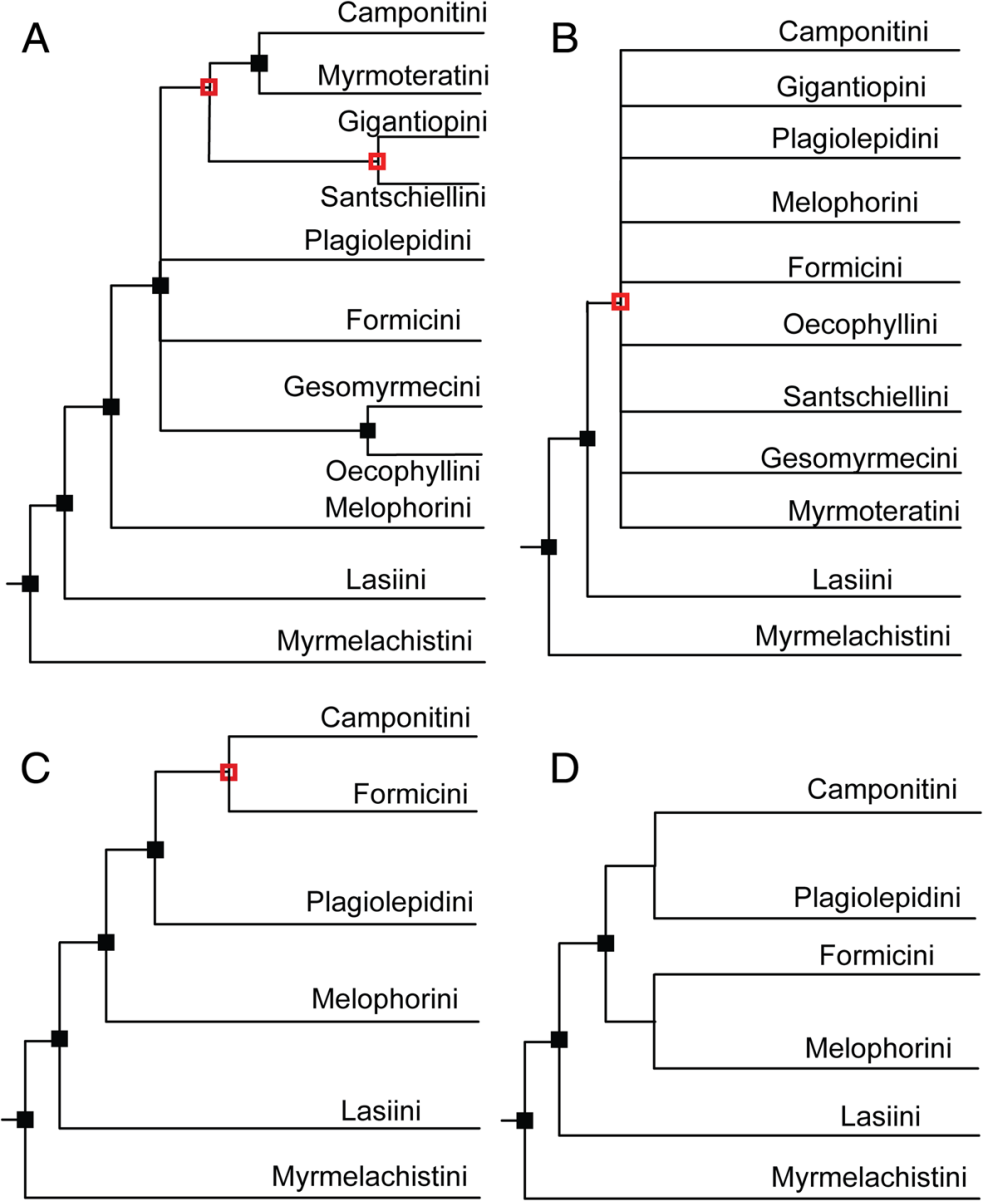
Comparison of support for major lineages within the Formicinae. Comparison of support for formicine tribes and the influence of the problematic taxa. Panel **a**) UCE-70 % data set, all taxa included; **b**) 10-gene data set, all taxa included; **c**) UCE-70 % data set, problematic genera excluded (*Santschiella, Gigantiops, Myrmoteras, Oecophylla, Gesomyrmex*); **d**) 10-gene data set, problematic genera excluded. Both figures are based on RAxML bootstrap searches, with 100 and 1152 replicates for UCE and 10-gene data set, respectively. See also Additional file 10

Overall, phylogenies resulting from maximum-likelihood analyses of each of our two main data sets (UCE-70 % and 10-gene data set) are congruent in topology for all parts of the phylogeny that receive high support, with disagreements restricted only to poorly resolved areas. The single exception is the position of *Myrmecocystus*. In the UCE-70 % phylogeny *Myrmecocystus* is sister to *Lasius*, whereas in the 10-gene data set this taxon arises within *Lasius*. The UCE-70 % phylogeny (Fig. 1a) is highly supported with only 12 (out of 85) nodes with BS < 100, whereas the 10-gene phylogeny (Fig. 1b), in contrast, retains 42 nodes with BS < 100. For example, generic relationships within the tribe Melophorini are well supported in the UCE tree, whereas these remain fairly unresolved in the 10-gene analysis. Interestingly, neither of the phylogenies resulting from the two data sets is able to fully resolve the relationships between the above-described major formicine lineages, i.e., both contain an ancient, unresolved polytomy. The UCE data set, however, provides substantially more resolution in this area of the phylogeny (Fig. 1a) than does the 10-gene phylogeny (Fig. 1b), reconstructing the Melophorini as sister to a clade containing the Camponotini, Plagiolepidini, and Formicini (in a polytomy). Figs. 2a&b provide contrasting summary sketches of the tribal relationships based on these two data sets. Bayesian analyses of the 10-gene data set produced very similar results (see Additional file 8). Maximum-likelihood analyses for the UCE-100best and 95 % data sets also show *Lasius* as paraphyletic, and overall phylogenies from these smaller UCE subsets are less well supported than from the full 70 % data set (see Additional file 9).

**Fig. 3:**
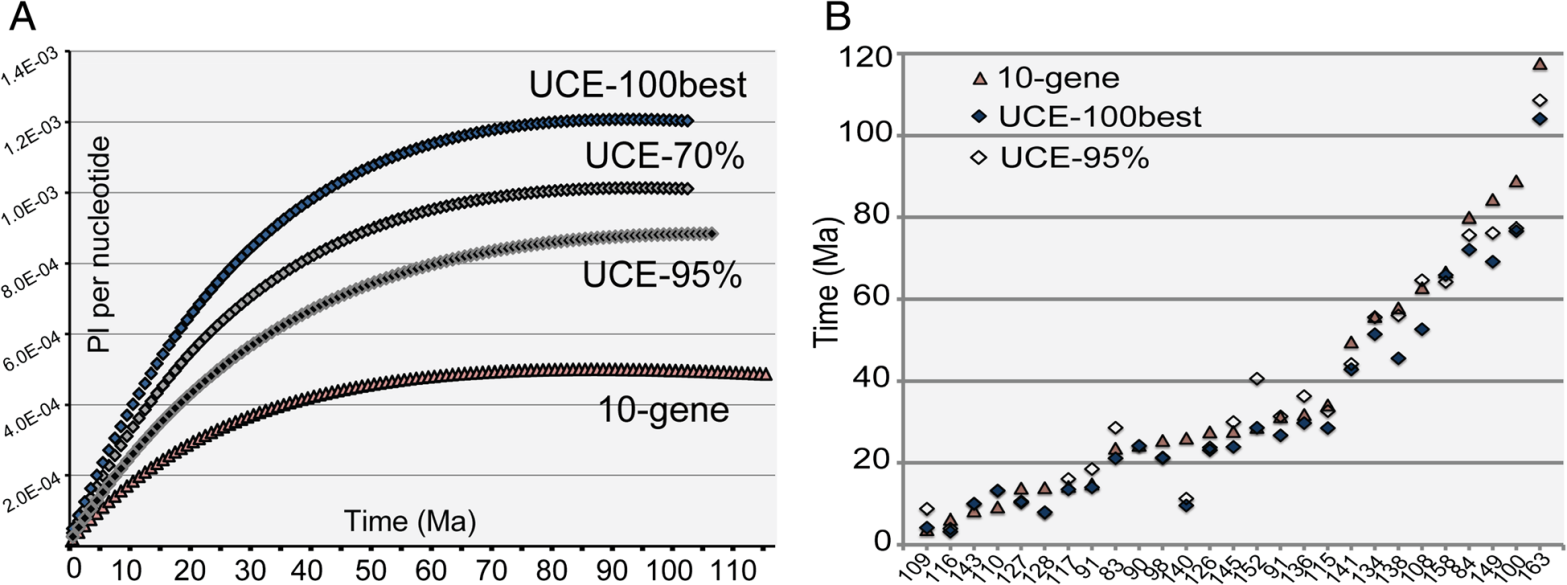
**a** Phylogenetic informativeness and **b**) comparison of divergence estimates. **a** Phylogenetic Informativeness (PI) as estimated with TAPIR [41] for the three UCE data sets and the 10-gene data set. PI is here plotted per nucleotide site as it increases with increasing age of divergence (in Ma) between taxa. **b** Graphic comparison of divergence time estimates for three BEAST analyses (UCE-100best, UCE-95 % and 10-gene data set); node labels correspond with those of Tables 2, Additional file 11, and Fig. 4

Phylogenetic informativeness (PI) increases in both data sets asymptotically with increasing divergence ages, but is much higher in the UCE data sets than in the 10-gene data set (Fig. 3a). The UCE-70 %, UCE-100best and UCE-95 % data sets show a 2.0-, 2.5- and 1.5-fold increase in PI relative to the 10-gene data set, respectively (Fig. 3a and Table 1).

### Taxa with uncertain relationships

We identified five taxa (*Gigantiops*, *Myrmoteras, Oecophylla*, *Gesomyrmex*, *Santschiella*) that are subtended by very long branches in phylogenies resulting from analyses of both data sets (Fig. 1). No analysis of either data set is decisively able to resolve the precise positions within the subfamily of *Gigantiops* or *Santschiella*. Strongly supported by the UCE but not by the ten-gene data, however, are a sister-group relationship between *Myrmoteras* and the tribe Camponotini (BS = 100) and a grouping of *Gesomyrmex* and *Oecophylla* as sister taxa (BS = 100).

We investigated the effect of these potential rogue taxa on tree topology, especially on the deep polytomy between subfamilies, and summarize results in Fig. 2 (see also Additional file 10). Excluding all five taxa resulted in a fully resolved, well-supported UCE phylogeny for the remaining six formicine lineages (Fig. 2c). This tree resolves the major polytomy with a relatively well-supported (BS = 93) sister-group relationship between Formicini and Camponotini, and with Plagiolepidini as the sister to (Formicini + Camponotini). In analyses of the 10-gene data set, in contrast, resolution of relationships between these tribes is only slightly improved by excluding the five problematic taxa (Fig. 2d).

### Divergence dating and biogeographic analyses

**Fig. 4:**
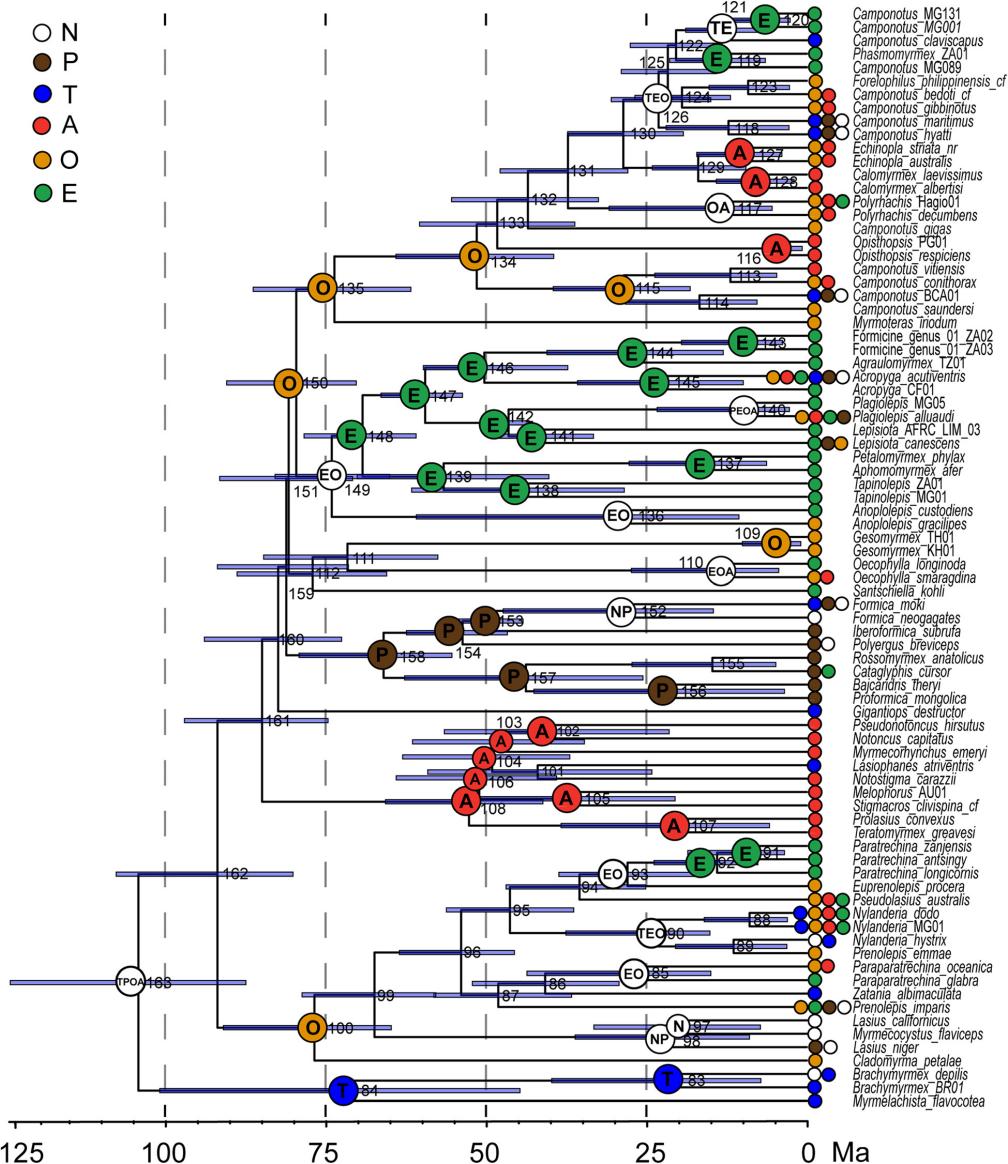
Time-calibrated phylogeny and ancestral range estimates for the subfamily Formicinae. Maximum clade credibility tree summarized from 48000 trees as estimated with the UCE-100best data set under a relaxed-clock model with nine fossil calibrations. Blue bars show the 95 % highest posterior density range for each node. Node numbers refer to Table 2 and Additional file 11. Ancestral ranges estimated by S-DEC are mapped on MRCA nodes for each tribe and genus (regardless of the level of support) and all other nodes that received high support (>70) for reconstructions. T = Neotropical, N = Nearctic, P = Palearctic, E = Afrotropical, O = Indomalayan and A = Australasian

With the exception of the positions of the five problematic or rogue taxa, analyses using BEAST produced results similar to those of other analyses with regard to topology. Figure 4 depicts the time-calibrated phylogeny as estimated from the UCE-100best data set, with the ancestral ranges estimated by the S-DEC model in RASP mapped onto each node. Support values, median crown group ages, select highest posterior density intervals (95 % HPD), and ancestral ranges are summarized in Table 2 (see also Additional file 11). Median age estimates and their 95 % HPD intervals are relatively similar across the three BEAST analyses, with ages differing by 15 MY at most (node 140, Fig. 3b and Table 2). Overall the two UCE data sets estimate slightly younger ages than the 10-gene data set. Ancestral range estimates under the two models (DEC and S-DEC) also mostly agree with each other (Fig. 4 and Table 2).

**Table 2:** Summary of crown group divergence ages and estimated ancestral ranges

	Node	PP	10 gene	UCE - 95 %	UCE - 100best	S-DEC		DEC	
						Area	Prob.	Area	Prob.
Formicinae	163	0.9	117.6 [100–136]	108.7 [92–127]	104.1 [88–125]	TPOA	19.0	TPEOA	22.0
Camponotini	134	1	55.8 [43–70]	55.6 [43–70]	51.4 [40–64]	O	43.4	O	45.5
Formicini	158	1	66.7 [55–80]	64.3 [54–75]	65.9 [55–79]	**P**	**89.9**	**P**	**100.0**
Lasiini	100	1	88.9 [72–106]	77.4 [65–92]	76.7 [65–91]	O	46.5	O	49.2
Melophorini	108	1	62.9 [43–85]	64.7 [49–84]	52.6 [41–66]	**A**	**99.9**	**A**	**100.0**
Myrmelachistini	84	1	80.0 [47–112]	75.7 [50–103]	72.2 [45–101]	**T**	**95.3**	**T**	**100.0**
Plagiolepidini	149	1	84.4 [71–99]	76.2 [66–88]	74.0 [65–83]	EO	52.9	E	51.2
*Camponotus s.s.*	126	1	27.6	23.8	23.1	TEO	26.9	PEO	26.7
*Colobopsis*	115	1	34.2	32.7	28.5	O	31.5	O	27.7
*Echinopla*	127	1	13.8	10.5	10.3	A	51.4	A	50.1
*Calomyrmex*	128	1	13.9	7.9	7.9	**A**	**100.0**	**A**	**100.0**
*Polyrhachis*	117	1	14.2	16.0	13.5	OA	38.1	OA	40.8
*Opisthopsis*	116	1	6.2	4.0	3.2	**A**	**100.0**	**A**	**100.0**
Formicine_genus01	143	1	8.3	9.9	10.0	**E**	**100.0**	**E**	**100.0**
*Tapinolepis*	138	1	57.88^a^	56.1	45.5	**E**	**100.0**	**E**	**100.0**
*Acropyga*	145	1	27.7	30.0	23.9	E	31.6	E	17.3
*Plagiolepis*	140	1	26.1	11.2	9.6	PEOA	48.7	PEOA	51.6
*Anoplolepis*	136	1	31.8	36.4	29.7	**EO**	**99.1**	**EO**	**100.0**
*Oecophylla*	110	1	9.2	13.2	13.2	**EOA**	**75.9**	**EOA**	**78.1**
*Gesomyrmex*	109	1	3.7	8.8	4.2	**O**	**100.0**	**O**	**100.0**
*Formica*	152	1	28.7	40.6	28.6	NP	68.2	**NP**	**71.8**
*Paratrechina*	92	1	14.8	18.5	14.0	**E**	**100.0**	**E**	**100.0**
*Nylanderia s.l.*	90	1	24.3	24.2	24.2	TEO	22.9	TEO	22.3
*Paraparatrechina*	85	1	31.3	31.4	26.8	EO	42.1	EO	40.4
*Lasius*	98	1	25.48^a^	21.18^a^	21.29^a^	**NP**	**76.5**	**NP**	**81.8**
*Brachymyrmex*	83	1	23.6	28.6	21.1	T	62.6	T	63.0
*Lepisiota*	141	0.93	49.51^a^	44.2	42.9	**E**	**99.7**	**E**	**100.0**

Table summarizing median crown group ages (in Ma, rounded to the first decimal) for selected formicine clades as estimated by BEAST analyses of different data sets. Bracketed numbers represent 95 % HPD (highest posterior density) intervals for selected nodes, rounded to the nearest integer. PP = posterior probability as estimated from the UCE-100best data set; ^a^ indicates this genus is not recovered as monophyletic in the particular analysis. Crown group ancestral ranges are further shown as estimated with the DEC and S-DEC models implemented in RASP for selected clades, bolded for probabilities > 75. Node numbers correspond to Fig. 3; only ranges with highest probability are shown. T = Neotropical, N = Neartic, P = Palearctic, E = Afrotropical, O = South-East Asian, A = Australian

Crown-group Formicinae are estimated to have evolved in the early Cretaceous, between 104.1–117.6 Ma. Ancestral range models estimate a very broad distribution range (TPOA/TPEOA; Table 2 and Fig. 4) for the most recent common ancestor (MRCA) of the Formicinae, although without much support. The six larger formicine tribes diversified throughout the late Cretaceous, Paleocene, and early Eocene, between 51–88.9 Ma (Fig. 4 and Table 2). The ancestral range analyses did not provide much support for ancient dispersal events (nodes 159–162) leading to the current distribution of these lineages, in accord with the uncertainty of phylogenetic relationships between them. The Lasiini are the oldest crown formicine lineage (76.7–88.9 Ma, node 100) and share an Indomalayan ancestor. Ancestral reconstructions and dispersal within this presently global lineage were not well supported. The sister group to all other Formicinae, the Myrmelachistini, is estimated to have a Neotropical origin between 72.2–80 Ma (node 84). Our analyses suggest that the Plagiolepidini evolved around the same time (76.7–88.9 Ma), but on a different continent: the Afrotropical and Indomalayan regions are reconstructed as ancestral ranges for crown-group Plagiolepidini. A Palearctic origin is further suggested for the Formicini in the Paleocene (64.3–66.7 Ma), while an early Eocene origin (51.4–55.8 Ma) of the Camponotini in the Indomalayan region received moderate support. Age estimates for crown-group Melophorini range from 52.6–62.9 Ma with an Australasian origin. For extant Formicinae genera, our crown-group estimates range from 3.2–56.1 Ma. Notably, the oldest genera within the Formicinae are *Lepisiota* (42.9–44.2 Ma, node 141) and *Tapinolepis* (45.5–56.1 Ma, node 138), while *Gesomyrmex* (4.2–8.8 Ma) and *Opisthopsis* (3.2–6.2 Ma) are recovered as the youngest lineages.

## Discussion

### Comparison of UCE vs multi-locus methods

We reconstruct the evolution of the subfamily Formicinae based upon a next-generation, pan-genomic data set of UCEs, and provide a direct comparison of this targeted-enrichment phylogenomic approach to a much smaller traditional phylogenetic data set assembled by Sanger-sequencing methods using the same set of 82 exemplar species. The Sanger data set was 100 % complete without missing data, while the UCE data set used for comparison was only 70 % complete. Our results clearly demonstrate the advantage of using the nearly 1000 UCE loci over using 10 genes to resolve formicine relationships. Only five nodes have less than 70 % bootstrap support in the UCE phylogeny (Fig. 1a), whereas 28 (out of 85) of the nodes in the 10-gene phylogeny are poorly supported (BS < 70). Such increased support in the UCE phylogeny compared to the 10-gene phylogeny is perhaps unsurprising, given the different scales of the data (~590000 bp vs ~7200 bp, Table 1). Furthermore, the superiority of the UCEs over the nuclear loci is not merely a function of sequence length, but can also be attributed to higher phylogenetic informativeness (PI). The full UCE-70 % data set has nearly double the PI relative to the 10-gene data set, while filtering of the UCE data by average bootstrap support (UCE-100best) raised PI to a level about 2.5 times higher. These metrics are congruent with estimates from a recent study comparing phylogenetic informativeness across ten single-copy nuclear genes with UCE core and flanking regions [8].

The remaining uncertainty in the UCE phylogeny could well be heavily influenced by the presence of the five problematic taxa subtended by long branches. Conversely, however, it should be stressed that although the exclusions of these taxa increase support for the remaining relationships, these exclusions could simultaneously lead to a decrease in phylogenetic accuracy due to less complete taxon sampling, and thus these results should not necessarily be interpreted as improved estimates of phylogenetic relationships (but see [47]).

### Dating with UCEs

To our knowledge, divergence ages based on UCEs have never been systematically compared to those estimated from other types of data, such as our ten-gene nuclear sequence data. It is possible that functional differences between these two types of data may lead to incompatible branch length estimation. All of our Sanger data were protein-coding or ribosomal DNA sequence from nuclear genes, whereas many UCE loci in general do not overlap with protein-coding regions, but rather appear to act as enhancers or splicing regulators [48]. Methods that jointly estimate divergence ages and tree topology, such as BEAST, have further seldom been employed to date with UCE or other genomic-scale data due to computational constraints. We overcame this limitation by filtering our data to a manageable size (i.e. 100 and 50 loci), and are thus able to compare for the first time age estimates derived from UCEs to those derived from our ten-gene nuclear data. For most nodes, the 10-gene data set estimated slightly older ages than the UCE data sets (Fig. 3b and Table 2). This is likely due to the high variance of evolutionary rates across loci included in the data sets, including the loci in the two different UCE data sets. Variance between estimates, however, is still much smaller than, for example, the 95 % HPC intervals around any of these age estimates (Table 2 and Fig. 4), suggesting that variance across loci is not the only factor influencing the differences in age estimates. We conclude that divergence dating with UCEs is both feasible and promising, and needs to be explored further as current methods and handling of genome-scale data sets continue to improve.

### Practical considerations of UCEs vs Sanger sequencing

While most researchers would agree that our results clearly show the advantage of using the nearly 1000 UCE loci over the 10-gene data set for phylogenetic inference, there are also practical aspects to consider regarding the cost and time spent in obtaining these data sets. We do not aim to provide a detailed analysis here, because both time and cost factors are highly variable and dependent on, e.g., sample DNA quality, available laboratory supplies, accumulated experience with a given technique, and sequencing cost at the respective genomic facility used. In our case, however, we found that the cost and time to generate both of these data sets are similar. Labor time associated with next-generation library preparation and target enrichment for UCEs for 90 taxa (~3 weeks for one full-time person) is roughly the same as for a single attempt at PCR-amplification and cycle sequencing of 10 individual genes, if not less. Processing time of the sequence data through the bioinformatics pipeline further is negligible compared to the time spent editing individual sequences. Cost of supplies and sequencing to generate ~1000 UCE loci can be as low as ~ $40–60/sample, compared to an estimate of $5/sample/gene fragment for PCR amplifications and Sanger sequencing. Thus, from our perspective, we found the UCE methodology comparable with regard to cost and time input and superior in terms of data output when compared to Sanger sequencing.

### Implications for formicine systematics

Based on our UCE phylogeny, we propose several taxonomic changes at the tribal level (see Additional file 7) for the subfamily Formicinae that aim to improve ant systematics while simultaneously keeping names fairly stable. These results partly agree with Bolton’s [49] prior system of formicine tribes based on morphology, although major changes have to be made in the compositions of Lasiini, Melophorini and Plagiolepidini, and the Myrmelachistini must be resurrected.

The five problematic taxa were previously unassociated with any of the larger clades, and to some extent this ambiguity persists. However, the UCE data firmly support the close relationship of *Gesomyrmex* and *Oecophylla*, and of *Myrmoteras* as the sister group of Camponotini; these relationships are poorly supported by the 10-gene data set. The phylogenetic positions of *Gigantiops* and *Santschiella* remain less clear, although the UCE data provide some support for a close relationship of these two taxa with Camponotini + *Myrmoteras*. Ancient radiation events are common throughout the insect tree of life [50], and other phylogenomic-scale studies have tried to resolve these with varying success ([e.g., [51–53]). Deep lineage diversification within the Formicinae appears to have occurred very rapidly, over a period of 10–12 MY in the Cretaceous (Fig. 4), and may challenge the information content of even phylogenomic data. In addition, although our sampling comprised representatives of nearly all extant formicine genera, our results could have been influenced by limited taxon sampling within these lineages, and thus increased taxon sampling may be able to improve phylogenetic resolution.

Our analyses recovered four formicine genera as non-monophyletic: *Nylanderia*, *Prenolepis*, *Lepisiota,* and *Camponotus*. Additional taxon sampling will be necessary to resolve the generic limits of the first three, although other unpublished data suggest that *Prenolepis emmae* may be misplaced in *Prenolepis* and actually belongs in *Nylanderia* (J. LaPolla, pers. comm.). We propose taxonomic changes here only for the carpenter ants (Ward et al., in review; see also Additional file 7), *Camponotus*, a genus for which paraphyly has been repeatedly indicated [16, 53–56]. Based on a strongly supported, well-sampled phylogeny, we resurrect the genera *Colobopsis* and *Dinomyrmex* for the two divergent lineages, and redefine *Camponotus* to include *Forelophilus* and *Phasmomyrmex*, thus making it monophyletic (Additional file 7). We found *Colobopsis* to be well separated from other *Camponotus* and sister to all other Camponotini, a result mirrored by phylogenetic analyses of their obligate bacterial endosymbionts, *Blochmannia*, unique to Camponotini [57]. The newly discovered sister relationship of *Myrmoteras* with Camponotini now raises the intriguing question of whether the former also harbor *Blochmannia* or related endosymbionts. Remarkably, we found the genera known to harbor the pumiliotoxins (*Brachymyrmex* and *Paratrechina*) sequestered by dendrobatid poison frogs [22] to be part of the two earliest branching lineages within the Formicinae, Myrmelachistini and Lasiini. This interesting pattern calls for a wider sampling and thorough investigation of these chemicals throughout the subfamily.

### Formicine biogeography

Our dating analyses extend formicine evolution deep into the Cretaceous (104.1–117.6 Ma). These median crown-group age estimates are considerably older than the fossil record suggests, with *Kyromyrma* (~92 Ma), the oldest known stem-group formicine fossil, relatively older than previous molecular dating estimates for the subfamily (77–83 Ma, [9]; 80–100 Ma, [10]; 75–90 Ma, [16]). The origin of the ant subfamily Myrmicinae was likewise recently estimated to be about 10 MY older than previous estimates [14]. Divergence dating analyses can be sensitive with regard to incorrectly placed fossil calibrations [58–60], but our analyses, sampling from the prior, show no indication of detrimental interactions between calibration priors. Another possibility is that an imbalance of ingroup vs. outgroup sampling and a lack of calibrations in the outgroup part of the phylogeny may be driving our age estimates, although we used outgroup taxa very similar to those in previous subfamily-level studies [11, 12, 14]. Conversely, our estimates may present a considerable improvement to previous studies for the very reason that our sampling of formicine lineages is more comprehensive.

The origin of the Formicinae was placed in the Neotropics by Moreau & Bell [16]. Our inference of a Neotropical origin for the Myrmelachistini—the oldest tribe and the sister lineage to the remaining formicines—agrees with this hypothesis. Further inference of biogeographic range evolution in the Formicinae was impeded in our study by the remaining phylogenetic uncertainty surrounding tribal relationships, but nonetheless we obtained highly supported crown-group ancestral range estimates for a number of lineages. The evolution of Melophorini took place mainly in Australasia (Fig. 4 and Table 2), which seems a natural result given that extant members of this tribe are largely confined to Australia. Along the same lines, the Formicini appeared to have had a history of evolution mainly in the Palearctic region, except for one dispersal to the Neotropics in the Eocene to Oligocene. For the Plagiolepidini, our analyses reconstructed an ancestral dispersal from the Oriental to the Afrotropical region (Fig. 3, node 150 to 151 to 148) where this tribe then appears to have undergone the majority of its diversification. Camponotini and Lasiini are two species-rich clades of formicine ants with representatives across all continents. For both of these globally distributed groups our estimates point to an origin in the Oriental region, although with mediocre support (Table 2, 43.4–45.5 % and 46.5–49.2 %). Moreau & Bell [16] have suggested that the Neotropics functioned as a cradle for ant diversification; however, our biogeographic results are not fully consistent with this hypothesis. While there are indications of a Formicinae origin in the Neotropics, our analyses overall do not associate the diversification of formicine ants with any particular region.

## Conclusions

We compared the phylogenetic informativeness of a 10-nuclear-gene data set produced by Sanger sequencing with a next-generation, phylogenomic data set of nearly 1000 UCE loci. This comparison, executed within the context of a case study of the same 82 species, tested the ability of these two types of data to resolve the evolutionary history of formicine ants. We found UCEs to be far superior to the multi-locus data set in estimating formicine relationships and noted a 1.5–2.5-fold increase in phylogenetic informativeness relative to the Sanger-produced data. Some ancient rapid divergence events remained unresolved even by our genomic data, indicating that phylogenetic reconstruction may in these cases only be improved with whole-genome data or, alternatively, that genuinely rapid radiations may have produced unresolvable hard polytomies. We successfully used BEAST to infer divergence ages from the UCE data, overcoming computational limitations through data filtering. These analyses reconstructed formicine ants and their major lineages to be relatively older compared to previous estimates for the group. The subfamily appears to have diversified across all biogeographic regions and to have had no particular evolutionary cradle, although much of the early history of the clade remains unclear. UCEs were able to significantly improve formicine tribal classification based on the comprehensive phylogeny for the group estimated here. Our study highlights both the promise and possible limitations of UCEs for evolutionary biologists considering the transition from Sanger to next-generation sequencing approaches: Taken together, our findings indicate UCEs are highly useful for insect phylogenomics. The resulting phylogeny reveals exciting foci for the study of behavior and chemical ecology in formicine ants.

## Availability of supporting data

The data sets supporting the results of this article are available in GenBank (Accessions KT443144–KT443783) and in the Sequence Read Archive (SUB1067415); data matrices and associated tree files are deposited in Treebase (TB2:S18146).

## Additional files

Additional file 1:
**Comprehensive description of methods.** An extensive version of the methods section, giving a more detailed account of laboratory and bioinformatics procedures. (PDF 132 kb) Additional file 2:
**Genbank accessions for PCR-amplified sequences included in this study.** Table including specimen identifiers and Genbank accession numbers for all study taxa. (PDF 94 kb) Additional file 3:
**Calibration points used to define prior calibration densities for dating analyses with BEAST.** (PDF 91 kb) Additional file 4:
**Taxon distribution matrix used in the biogeographic analyses.** (PDF 81 kb) Additional file 5:
**Dispersal constraint matrices used for DEC and S-DEC analyses in RASP.** (PDF 75 kb) Additional file 6:
**Summary of UCE capture statistics.** (PDF 95 kb) Additional file 7:
**Revised tribal classification for the Formicinae.** (PDF 84 kb) Additional file 8:
**Phylogenetic trees from analyses not illustrated in the main text.** Additional results from Bayesian and Maximum Likelihood bootstrap analyses. (PDF 2225 kb) Additional file 9:
**Phylogenetic trees from analyses not illustrated in the main text, continued.** Additional results from Maximum Likelihood analyses on UCE data subsets. (PDF 669 kb) Additional file 10:
**Summary of taxon exclusion experiments.** Summary sketches of phylogenetic relationships contrasting the placement of the seven rogue taxa between UCE and 10-gene data set. (PDF 248 kb) Additional file 11:
**Comprehensive results of ancestral range estimations under the DEC and S-DEC models.** Extended version of Table 2 including all crown group ancestral ranges as estimated with the DEC and S-DEC models implemented in RASP. (PDF 97 kb)

## Abbreviations

BI: Bayesian inference
BS: Bootstrap support
bp: base pairs
DEC: Dispersal-extinction-cladogenesis
HPD: Highest posterior density
Ma: Million years ago
MCC: Maximum clade credibility
ML: Maximum Likelihood
MRCA: Most recent common ancestor
MY: Million years
PI: Phylogenetic informativeness
PP: Posterior Probability
S-DEC: Statistical Dispersal-extincton-cladogenesis
SI/HPC: Smithsonian Institution high performance cluster
UCEs: Ultraconserved elements

## References

[1] McCormack JE, Faircloth BC, Crawford NG, Gowaty PA, Brumfield RT, Glenn TC (2012). Ultraconserved elements are novel phylogenomic markers that resolve placental mammal phylogeny when combined with species-tree analysis. Gen Res.

[2] Lemmon EM, Lemmon AR (2013). High-throughput genomic data in systematics and phylogenetics. Annu Rev Ecol Syst.

[3] Smith BT, Harvey MG, Faircloth BC, Glenn TC, Brumfield RT (2013). Target capture and massively parallel sequencing of ultraconserved elements for comparative studies at shallow evolutionary time scales. Syst Biol.

[4] Smith BT, McCormack JE, Cuervo AM, Hickerson MJ, Aleixo A, Cadena CD, Perez-Eman J, Burney CW, Xie X, Harvey MG (2014). The drivers of tropical speciation. Nature.

[5] Crawford NG, Faircloth BC, McCormack JE, Brumfield RT, Winker K, Glenn TC (2012). More than 1000 ultraconserved elements provide evidence that turtles are the sister group of archosaurs. Biol Lett.

[6] Faircloth BC, McCormack JE, Crawford NG, Harvey MG, Brumfield RT, Glenn TC (2012). Ultraconserved elements anchor thousands of genetic markers spanning multiple evolutionary timescales. Syst Biol.

[7] Faircloth BC, Branstetter MG, White ND, Brady SG (2015). Target enrichment of ultraconserved elements from arthropods provides a genomic perspective on relationships among Hymenoptera. Mol Ecol Res.

[8] Gilbert PS, Chang J, Pan C, Sobel EM, Sinsheimer JS, Faircloth BC, Alfaro ME (2015). Genome-wide ultraconserved elements exhibit higher phylogenetic informativeness than traditional gene markers in percomorph fishes. Mol Phylogenet Evol.

[9] Brady SG, Schultz TR, Fisher BL, Ward PS (2006). Evaluating alternative hypotheses for the early evolution and diversification of ants. Proc Natl Acad Sci.

[10] Moreau CS, Bell CD, Vila R, Archibald SB, Pierce NE (2006). Phylogeny of the ants: Diversification in the age of angiosperms. Science.

[11] Ward PS, Brady SG, Fisher BL, Schultz TR (2010). Phylogeny and biogeography of dolichoderine ants: effects of data partitioning and relict taxa on historical inference. Syst Biol.

[12] Brady SG, Fisher BL, Schultz TR, Ward PS (2014). The rise of army ants and their relatives: diversification of specialized predatory doryline ants. BMC Evol Biol.

[13] Schmidt CA, Shattuck SO (2014). The higher classification of the ant subfamily Ponerinae (Hymenoptera: Formicidae), with a review of ponerine ecology and behavior. Zootaxa.

[14] Ward PS, Brady SG, Fisher BL, Schultz TR (2015). The evolution of myrmicine ants: phylogeny and biogeography of a hyperdiverse ant clade (Hymenoptera: Formicidae). Syst Ent.

[15] Lucky A, Trautwein MD, Guenard BS, Weiser MD, Dunn RR (2013). Tracing the rise of ants-out of the ground. PLoS ONE.

[16] Moreau CS, Bell CD (2013). Testing the museum versus cradle tropical biological diversity hypothesis: phylogeny, diversification, and ancestral biogeographic range evolution of the ants. Evolution.

[17] Blaimer BB, Brady SG, Schultz TR, Fisher BL (2015). Functional and phylogenetic approaches reveal the evolution of diversity in a hyper diverse biota. Ecography.

[18] Schultz TR, Brady SG (2008). Major evolutionary transitions in ant agriculture. Proc Natl Acad Sci.

[19] Price SL, Powell S, Kronauer DJC, Tran LAP, Pierce NE, Wayne RK (2014). Renewed diversification is associated with new ecological opportunity in the Neotropical turtle ants. J Evol Biol.

[20] Ward PS (2014). The phylogeny and evolution of ants. Annu Rev Ecol Syst.

[21] Schmidt JO. Chemistry, pharmacology, and chemical ecology of ant venoms. In: Piek T, editor. Venoms of the Hymenoptera*.* London: Academic Press; 1986: 425–508

[22] Saporito RA, Garraffo HM, Donnelly MA, Edwards AL, Longino JT, Daly JW (2004). Formicine ants: An arthropod source for the pumiliotoxin alkaloids of dendrobatid poison frogs. Proc Natl Acad Sci.

[23] Buschinger A (2009). Social parasitism among ants: a review (Hymenoptera: Formicidae). Myrmecol News.

[24] LaPolla JS, Brady SG, Shattuck SO (2010). Phylogeny and taxonomy of the *Prenolepis* genus-group of ants (Hymenoptera: Formicidae). Syst Ent.

[25] Lapolla JS, Kallal RJ, Brady SG (2012). A new ant genus from the Greater Antilles and Central America, *Zatania* (Hymenoptera: Formicidae), exemplifies the utility of male and molecular character systems. Syst Ent.

[26] Blumenstiel B, Cibulskis K, Fisher S, DeFelice M, Barry A, Fennell T, et al. Targeted exon sequencing by in-solution hybrid selection. Curr Protoc Hum Genet. 2010;Unit 18.4.1–18.4.24, Supplement 66. 10.1002/0471142905.hg1804s66 20582916

[27] Ward PS, Downie DA (2005). The ant subfamily Pseudomyrmecinae (Hymenoptera: Formicidae): phylogeny and evolution of big-eyed arboreal ants. Syst Ent.

[28] Ward PS, Sumnicht TP (2012). Molecular and morphological evidence for three sympatric species of *Leptanilla* (Hymenoptera: Formicidae) on the Greek island of Rhodes. Myrmecol News.

[29] Katoh K, Asimenos G, Toh H. Multiple alignment of DNA sequences with MAFFT. In: Bioinformatics for DNA sequence analysis. Humana Press, New York City; 2009: 39–64 10.1007/978-1-59745-251-9_3 19378139

[30] Faircloth B. Illumiprocessor: a trimmomatic wrapper for parallel adapter and quality trimming. 2013. doi: 10.6079/J9ILL

[31] Bolger AM, Lohse M, Usadel B. Trimmomatic: a flexible trimmer for Illumina sequence data. Bioinformatics 2014; btu170:1–7. 10.1093/bioinformatics/btu170 PMC4103590 24695404

[32] Faircloth B. PHYLUCE is a software package for the analysis of conserved genomic loci. 2015. doi:10.6079/J9PHYL 10.1093/bioinformatics/btv646 26530724

[33] Grabherr MG, Haas BJ, Yassour M, Levin JZ, Thompson DA, Amit I, Adiconis X, Fan L, Raychowdhury R, Zeng Q (2011). Full-length transcriptome assembly from RNA-Seq data without a reference genome. Nat Biotech.

[34] Castresana J (2000). Selection of conserved blocks from multiple alignments for their use in phylogenetic analysis. Mol Biol Evol.

[35] Lanfear R, Calcott B, Ho SYW, Guindon S (2012). PartitionFinder: Combined selection of partitioning schemes and substitution models for phylogenetic analyses. Mol Biol Evol.

[36] Stamatakis A (2006). RAxML-VI-HPC: maximum likelihood-based phylogenetic analyses with thousands of taxa and mixed models. Bioinformatics.

[37] Zwickl DJ. Genetic algorithm approaches for the phylogenetic analysis of large biological sequence datasets under the maximum likelihood criterion. The University of Texas at Austin; 2006; accessible at: https://repositories.lib.utexas.edu/handle/2152/2666

[38] Ronquist F, Teslenko M, van der Mark P, Ayres DL, Darling A, Höhna S, Larget B, Liu L, Suchard MA, Huelsenbeck JP (2012). MrBayes 3.2: efficient Bayesian phylogenetic inference and model choice across a large model space. Syst Biol.

[39] Frandsen PB, Calcott B, Mayer C, Lanfear R (2015). Automatic selection of partitioning schemes for phylogenetic analyses using iterative k-means clustering of site rates. BMC Evol Biol.

[40] Townsend JP (2007). Profiling Phylogenetic Informativeness. Syst Biol.

[41] Faircloth BC, Chang J, Alfaro ME. TAPIR enables high-throughput estimation and comparison of phylogenetic informativeness using locus-specific substitution models. 2012. arXiv preprint arXiv:12021215.

[42] López-Giráldez F, Townsend JP (2011). PhyDesign: an online application for profiling phylogenetic informativeness. BMC Evol Biol.

[43] Drummond AJ, Suchard MA, Xie D, Rambaut A (2012). Bayesian Phylogenetics with BEAUti and the BEAST 1.7. Mol Biol Evol.

[44] Ree RH, Smith SA (2008). Maximum likelihood inference of geographic range evolution by dispersal, local extinction, and cladogenesis. Syst Biol.

[45] Beaulieu JM, Tank DC, Donoghue MJ (2013). A Southern Hemisphere origin for campanulid angiosperms, with traces of the break-up of Gondwana. BMC Evol Biol.

[46] Yu Y, Harris AJ, Blair C, He X (2015). RASP (Reconstruct Ancestral State in Phylogenies): a tool for historical biogeography. Mol Phylogen Evol.

[47] Aberer AJ, Krompass D, Stamatakis A (2013). Pruning rogue taxa improves phylogenetic accuracy: an efficient algorithm and webservice. Syst Biol.

[48] Makunin IV, Shloma VV, Stephen SJ, Pheasant M, Belyakin SN (2013). Comparison of ultra-conserved elements in Drosophilids and Vertebrates. PLoS ONE.

[49] Bolton B (2003). Synopsis and classification of Formicidae. Mem Am Entomol Inst.

[50] Whitfield JB, Kjer KM (2008). Ancient rapid radiations of insects: challenges for phylogenetic analysis. Ann Rev Ent.

[51] Wiegmann BM, Trautwein MD, Winkler IS, Barr NB, Kim J-W, Lambkin C, Bertone MA, Cassel BK, Bayless KM, Heimberg AM (2011). Episodic radiations in the fly tree of life. Proc Natl Acad Sci.

[52] Bazinet AL, Cummings MP, Mitter KT, Mitter CW. Can RNA-Seq resolve the rapid radiation of advanced moths and butterflies (Hexapoda: Lepidoptera: Apoditrysia)? An exploratory study. PLoS ONE. 2013; 8(12):e82615. 10.1371/journal.pone.0082615 PMC3853519 24324810

[53] Johnson BR, Borowiec ML, Chiu JC, Lee EK, Atallah J, Ward PS (2013). Phylogenomics resolves evolutionary relationships among ants, bees, and wasps. Curr Biol.

[54] Brady SG, Gadau J, Ward PS, Austin AD, Dowton M (2000). Systematics of the ant genus *Camponotus* (Hymenoptera: Formicidae): a preliminary analysis using data from the mitochondrial gene cytochrome oxidase I. Hymenoptera: evolution, biodiversity and biological control.

[55] Chen Z, Zhou S, Ye D, Chen Y, Lu C (2013). Molecular phylogeny of the ant subfamily Formicinae (Hymenoptera, Formicidae) from China based on mitochondrial genes. Sociobiology.

[56] Williams LE, Wernegreen JJ (2015). Genome evolution in an ancient bacteria-ant symbiosis: parallel gene loss among *Blochmannia* spanning the origin of the ant tribe Camponotini. PeerJ.

[57] Wernegreen JJ, Kauppinen SN, Brady SG, Ward PS (2009). One nutritional symbiosis begat another: phylogenetic evidence that the ant tribe Camponotini acquired *Blochmannia* by tending sap-feeding insects. BMC Evol Biol.

[58] Near TJ, Sanderson MJ (2004). Assessing the quality of molecular divergence time estimates by fossil calibrations and fossil-based model selection. Philos Trans R Soc Lond B Biol Sci.

[59] Ho SYW, Phillips MJ (2009). Accounting for calibration uncertainty in phylogenetic estimation of evolutionary divergence times. Syst Biol.

[60] Brady SG (2011). Effects of fossil calibration-uncertainty on divergence dating in ants and bees. Am Ent.

