# Supplementary material for: Phylogenomic methods outperform traditional multi-locus approaches in resolving deep evolutionary history: a case study of formicine ants
Source: BMC Evol Biol. 2015 Dec 4;15:271. doi: 10.1186/s12862-015-0552-5 (PMC4670518; doi:10.1186/s12862-015-0552-5)
Supplement: Additional file 1: — Comprehensive description of methods. An extensive version of the methods section, giving a more detailed account of laboratory and bioinformatics procedures. (PDF 132 kb) [file 12862_2015_552_MOESM1_ESM.pdf]

**Additional file 1**: Comprehensive description of methods

### Molecular data collection

*Taxon sampling and DNA extractions*. Our data set comprised 90 ant species, 82 of which represent 48 of the 51 currently-valid formicine genera. We further included eight outgroup taxa from other seven ant subfamilies (Myrmicinae, Ectatomminae, Heteroponerinae, Pseudomyrmecinae, Myrmeciinae, Aneuretinae, Dolichoderinae) belonging to the formicoid clade of ants (sensu Brady et al. [1]), and trees were rooted using the four most distantly related subfamilies to the formicines. For some more speciose groups, multiple species per genus were selected to represent major lineages and to resolve taxonomic questions. Specimens included in this study were collected in accordance with local regulations and all necessary permits were obtained. Voucher specimens have been deposited at the University of California, Davis, at the National Museum of Natural History, and at the California Academy of Sciences, with specimen data available on AntWeb (www.antweb.org); see Additional file 2 for specimen identifiers. DNA was extracted destructively or non-destructively (specimen retained after extraction) from worker ants or pupae using a DNeasy Blood and TissueKit (Qiagen, Valencia, CA, USA).

*Library preparation, target enrichment and sequencing of UCEs*. We quantified DNA for each sample using a Qubit fluorometer (High sensitivity kit, Life Technologies, Inc.) and sheared 2.8–497 ng (139 ng mean) DNA to a target size of approximately 500–600 bp by sonication (Q800 or Diagenode BioRuptor; Qsonica Inc.). The sheared DNA was used as input for a modified genomic DNA library preparation protocol (Kapa Hyper Prep Library Kit, Kapa Biosystems) that incorporated "with-bead" cleanup steps [2] and a generic SPRI substitute [3], "speedbeads" hereafter), as described by [4]. We used TruSeq-style adapters during adapter ligation [5], and

PCR amplified 50% of the resulting library volume (15 µL) using a reaction mix of 25 µL HiFi HotStart polymerase (Kapa Biosystems), 2.5 µL each of Illumina TruSeq-style i5 and i7 primers (5 µM each) and 5 µL double-distilled water (ddH20). We used the following thermal protocol: 98 ºC for 45 s; 13 cycles of 98 ºC for 15 s, 65 ºC for 30 s, 72 ºC for 60 s, and final extension at 72 ºC for 5 m. After rehydrating (in 23µL pH 8 Elution Buffer (EB hereafter)) and purifying reactions using 1.0X speedbeads, we combined groups of eight libraries at equimolar ratios into enrichment pools having final concentrations of 74–156 ng/µL.

We enriched each pool using a set of 2749 custom-designed probes (MYcroarray, Inc.) targeting 1510 UCE loci in Hymenoptera (see [4]). We followed library enrichment procedures for the MYcroarray MYBaits kit [6], except we used a 0.1X concentration of the standard MYBaits concentration, and added 0.7 µL of 500 µM custom blocking oligos designed against our custom sequence tags. We ran the hybridization reaction for 24 h at 65 °C, subsequently bound all pools to streptavidin beads (MyOne C1; Life Technologies), and washed bound libraries according to a standard target enrichment protocol [6]. We used the with-bead approach for PCR recovery of enriched libraries as described in Faircloth et al. [4]. We combined 15 µL of streptavidin bead-bound, enriched library with 25 µL HiFi HotStart Taq (Kapa Biosystems), 5 µL of Illumina TruSeq primer mix (5 µM each) and 5 µL of ddH2O. We ran post-enrichment PCR using the following thermal profile: 98 °C for 45 s; 18 cycles of 98 °C for 15 s, 60 °C for 30 s, 72 °C for 60 s; and a final extension of 72 °C for 5 m. We purified resulting reactions using 1.0X speedbeads, and we rehydrated the enriched pools in 22 µL EB. We quantified 2 µL of each enriched pool using a Qubit fluorometer (broad range kit).

Enrichment was verified by amplifying seven UCE loci (for primers see [4]) targeted by the probe set. We set up a relative qPCR by amplifying two replicates of 1 ng of enriched DNA from

each library at all seven loci and comparing those results to two replicates of 1 ng unenriched DNA for each library at all seven loci. We performed qPCR using a SYBR® FAST qPCR kit (Kapa Biosystems) on aViiATM 7 (Life Technologies). Following data collection, we computed the average of the replicate crossing point (Cp) values for each library at each amplicon, and we computed fold-enrichment values, assuming an efficiency of 1.78 and using the formula 1.78abs(enriched Cp−unenriched Cp). We then created serial dilutions of each pool (1:200,000, 1:800,000, 1:1.000,000, 1:10.000,000) and performed qPCR library quantification, assuming an average library fragment length of 600 bp. Based on the size-adjusted concentrations estimated by qPCR, we pooled libraries at equimolar concentrations and size-selected for 250–800 with a BluePippin (SageScience). The pooled libraries were sequenced using two partial lanes (both lanes included samples from other projects) of a 150-bp paired-end Illumina HiSeq 2500 run (U Cornell Genomics Facility). All of the UCE laboratory work was conducted in and with support of the Laboratories of Analytical Biology (L.A.B.) facilities of the National Museum of Natural History. Quality-trimmed sequence reads generated as part of this study are available from the NCBI Sequence Read Archive (SUB1067415).

*Amplification, Sanger sequencing, and alignment of nuclear loci*. Ten nuclear markers commonly used in ant systematics were selected for amplification [for primers see 1, 7, 8, 9] Long-wavelength rhodopsin (*LW Rh*, 458 bp), elongation factor 1-alpha F1 (*EF1aF1*, 359 bp), elongation factor 1-alpha F2 (*EF1aF2*, 517bp), abdominal-A (*abdA*, 606bp), arginine kinase (*argK*, 673bp), ultrabithorax (*Ubx*, 630bp),18S ribosomal DNA (rDNA, 1851 bp), 28S rDNA (825 bp), wingless (*Wg*, 412 bp) and topoisomerase 1 (*Top1*, 883 bp), for a total of 7214 bp in the aligned data matrix. Amplifications were performed using standard PCR methods outlined in

Ward and Downie [7] and cycle sequencing reactions were performed using PCR primers and BigDye ® Terminator ver. 3.1 Cycle Sequencing chemistry. Amplicons were analyzed on ABI 3730 Sequencers © (2011 Life Technologies, Frederick, MA) housed at the College of Biological Sciences DNA Sequencing Facility, University of California, Davis, CA and at L.A.B. at the National Museum of Natural History (NMNH), Washington, DC. Sequence data were assembled and edited in Sequencher v5 (Gene Codes Corporation, Ann Arbor, MI) and aligned with MAFFT v7.017 [10]. All newly generated sequences have been deposited in Genbank, under accessions KT443144–KT443783 (see Additional file 2).

**Processing and alignment of UCE data**

We trimmed the demultiplexed FASTQ data output for adapter contamination and low-quality bases using Illumiprocessor [11], based on the package Trimmomatic [12]. All further data processing described in the following relied on scripts within the PHYLUCE package ([13], but see also [14]). We computed summary statistics on the data using the get_fastq_stats.py script, and assembled the cleaned reads using the assemblo_trinity.py wrapper around the program Trinity (version trinityrnaseq_r20140717) [15]. Average sequencing coverage across assembled contigs was calculated using get_trinity_coverage.py.

To identify assembled contigs representing enriched UCE loci from each species, species-specific contig assemblies were aligned to a FASTA file of all enrichment baits using match_contigs_to_probes.py. (min_coverage=50, min_identity=80), and sequence coverage statistics (avg, min, max) for contigs containing UCE loci were calculated using get_trinity_coverage_for_uce_loci.py. Subsequently, we used get_match_counts.py to query the

relational database containing matched probes created in the previous step, in order to generate a list of UCE loci shared across all taxa. This list of UCE loci was then used in the get_fastas_from_match_counts.py script to create FASTA files for each UCE locus, which contain sequence data for taxa present at that particular locus. We aligned all data in all these FASTA files using MAFFT [10] through seqcap_align_2.py (min-length=20, no-trim). Following alignment, we further trimmed our alignment using a wrapper script (get_gblocks_trimmed_alignment_from _untrimmed.py) for Gblocks [16] using the following settings: b1=0.5, b2=0.5, b3=12, b4=7.

We initially selected the following subsets of UCE alignments using get_only_loci_with_min_taxa.py: 1) 50% complete (containing alignment data from at least 45 of the 90 taxa), 2) 60% complete (>54 of 90 taxa), 3) 70% complete (>63 of 90 taxa) and 4) 95% (>85 of 90 taxa). We added missing data designators to each file with add_missing_data_designators.py, and generated alignment statistics across all alignments using get_align_summary_data.py. Finally, we concatenated individual alignments of UCE loci for each subset into one nexus alignment file with format_nexus_files_for_raxml.py for subsequent phylogenetic analyses.

**Phylogenetic inference**

For the Sanger-sequenced, 10-gene data set, PartitionFinder v.1.1.1 [17] was used to simultaneously select data partitions and estimate appropriate models of evolution. The 10-gene data set was analyzed using standard Bayesian and maximum likelihood (ML) methods. Maximum likelihood analyses were carried out in the programs RAxML v7.7.7 [18] and GARLI

v.2.0 [19] and comprised best tree (5 runs per search; number of searches GARLI=100, RAxML=192) and bootstrap searches (GARLI N=1000, RAxML N=1152). Bayesian inference was performed in MRBAYES 3.2 [20] with 2 independent runs of 40 million generations each; MCMC convergence was checked visually with TRACER v1.6 (http://tree.bio.ed.ac.uk/software/tracer/) and by examining PSRF values in MrBayes .stat output files. 72000 trees were summarized by a consensus tree after discarding a burnin of 10%. All analyses were carried out using parallel processing (one chain per CPU) on a 12-core Intel-processor Apple computer or on the Smithsonian NMNH LAB Topaz network of Apple computers with Intel processors.

For the UCE phylogenomic data set, a development version of PartitionFinder [21] was used to select data partitions, which depends on the software fast_TIGER (http://dx.doi.org/10.5281/zenodo.12914) and is designed to handle large genome-scale data sets. The UCE data set was analyzed with ML methods only, given the current computational limits of Bayesian inference for large data sets. We performed ML best tree and bootstrap searches (N=100) in RAxML v8.0.3 [18], initially on a 50%, 60%, 70% and 95% complete UCE matrix (see above). For subsequent analyses, however, we elected to proceed with the 70% and 95% matrices. We also reconstructed gene trees for the 959 UCE loci in the 70% matrix by performing RAxML analyses (best tree and bootstrap) on individual loci. The results were then used for a variety of filtering experiments in order to reduce the size of the data set for downstream analyses. Based on calculations of average branch lengths and average bootstrap support for each gene tree in R (scripts by M. Borowiec available at https://github.com/marekborowiec/metazoan_phylogenomics/blob/master/gene_stats.R) we filtered the data into four subsets: 1) 100 loci with the shortest average branch lengths, 2) 100

loci with the longest average branch lengths, 3) 100 loci with the highest average bootstrap score and 4) 50 loci with the highest average bootstrap score. We chose the data set representing the 100 loci with the best average bootstrap score (3, UCE-100best hereafter), for further analyses. The four main data sets used for downstream analyses are summarized in Table 1. We calculated phylogenetic informativeness (PI) [22] per nucleotide site for these three UCE and the 10-gene data set with the software package TAPIR [23] (http://faircloth-lab.github.com/tapir/), a parallelized version of PhyDesign [24]. PI can be described as a function of the evolutionary rate and the divergence time to most recent common ancestor among the taxa under analysis and estimates the probability that a character resolves a hypothetical polytomy in a four-taxon phylogeny [22, 25].

We identified five long-branched taxa influencing resolution in all our analyses (UCE data sets and 10-gene data set) and changing positions in the phylogenies resulting from different data sets and analyses. In order to better understand the effects of these taxa on phylogenetic results, we carried out phylogenetic analyses (BI for 10-gene, ML for UCEs) with a series of taxon-reduced data sets. These analyses excluded all or various combinations of the following taxa: *Gesomyrmex_TH01*, *Gesomyrmex_KH01*, *Santschiella kohli*, *Oecophylla longinoda*, *Oecophylla smaragdina*, *Myrmoteras iriodum*, *Gigantiops destructor*. All of the above phylogenetic analyses were performed on the Smithsonian Institution high performance cluster (SI/HPC). Data matrices as well as the resulting tree files for the four main data sets are deposited in Treebase (TB2:S18146 ).

## Dating analyses and ancestral range reconstructions

We inferred divergence dates within the Formicinae from both the our UCE-100best, UCE-95%, and the 10-gene data sets with the program BEAST v1.8 [43],run via the SI/HPC. We performed analyses on both a partitioned and unpartitioned version (results not shown) of the 10-gene data set, consisting of four independent runs with a chain length of 500 million generations (see Table 1). The UCE analyses consisted of two runs each for 95% and 100best data sets, with a chain length of 300 million generations. We also attempted to use an unpartitioned version of the full UCE-70% data set for BEAST analyses, but were not able to reach convergence in those analyses. All divergence analyses were calibrated by placing calibration priors on nine nodes in the phylogeny (see Additional file 3), and we checked the performance of these priors by also performing the analysis without data, sampling only from the prior ("empty"). We employed a diffuse gamma distribution on the mean branch lengths (ucld.mean;alpha=0.001, beta=1000), but otherwise left all remaining priors at their default values. Trace files were analyzed in Tracer v1.6 to determine chain convergence and burnin. Tree files were then summarized with LogCombiner v1.8.2 and TreeAnnotator v1.8.2 after discarding a burnin of 20%.

## Biogeographic analyses

We constructed a species distribution matrix to evaluate the biogeographic history of Formicinae as follows (see Additional file 5). We assigned to each terminal taxon the distribution of its species plus that of other species estimated to be more closely related to the terminal taxon than to any other species in our data set. For example, the Nearctic species *Formica moki* was coded

as occupying the Neotropical and Palearctic regions, in addition to the Nearctic, in recognition of other species of the *Formica fusca*-group occurring in those regions [27]. This protocol helps to reduce the influence of missing taxa (and their range information) on our ancestral range inferences. We used the dispersal-extinction-cladogenesis model (DEC, "Lagrange," [28]) and the statistical DEC model (S-DEC, "Bayes-Lagrange", [29]) implemented in the program RASP [30] to estimate ancestral ranges. The main difference between these two methods is that DEC performs estimations on a condensed tree (i.e. a BEAST MCC tree), whereas S-DEC optimizes estimations over a set of trees and thus takes topological uncertainty into account. We used the resulting set of trees and the respective MCC tree from our BEAST analysis on the UCE-100best data set for these biogeographic analyses. For S-DEC, 1000 trees were randomly chosen from the set of 48000 trees. Under both models, outgroups were removed before the analyses. We followed Ward et al. [31] in designating six biogeographic areas (Neotropical, Nearctic, Palearctic, Afrotropical, Indomalayan and Australasian) and defined different dispersal constraints for two time slices (0–50 Ma and 50–105 Ma) based on paleogeography (Scotese, 2010, PALEOMAP project; http://www.scotese.com/ ) (see Additional file 5). The maximum number of possible areas for each state was set=6 and we included all possible range combinations in the analyses.

**References**


1. Brady SG, Schultz TR, Fisher BL, Ward PS: **Evaluating alternative hypotheses for the early evolution and diversification of ants**. *Proc Natl Acad Sci* 2006, **103**:18172–18177.
2. Fisher S, Barry A, Abreu J, Minie B, Nolan J, Delorey TM, Young G, Fennell TJ, Allen A, Ambrogio L: **A scalable, fully automated process for construction of sequence-ready human exome targeted capture libraries**. *Genome Biol* 2011, **12**:R1.
3. Rohland N, Reich D: **Cost-effective, high-throughput DNA sequencing libraries for multiplexed target capture**. Genome Res 2012, **22**:939–946.
4. Faircloth BC, Branstetter MG, White ND, Brady SG: **Target enrichment of ultraconserved elements from arthropods provides a genomic perspective on relationships among Hymenoptera**. *Mol Ecol Res* 2015, **15**:489–501.
5. Faircloth BC, Glenn TC: **Not all sequence tags are created equal: designing and validating sequence identification tags robust to indels**. *PLoS ONE* 2012, **7**:e42543.
6. Blumenstiel B, Cibulskis K, Fisher S, DeFelice M, Barry A, Fennell T, Abreu J, Minie B, Costello M, Young G: **Targeted exon sequencing by in-solution hybrid selection**. *Curr Protoc Hum Genet* 2010:166.
7. Ward PS, Downie DA: **The ant subfamily Pseudomyrmecinae (Hymenoptera: Formicidae): phylogeny and evolution of big-eyed arboreal ants**. *Syst Ent* 2005, **30**:310–335.
8. Ward PS, Brady SG, Fisher BL, Schultz TR: **Phylogeny and biogeography of dolichoderine ants: effects of data partitioning and relict taxa on historical inference**. *Syst Biol* 2010, **59**:342–362.
9. Ward PS, Sumnicht TP: **Molecular and morphological evidence for three sympatric species of *Leptanilla* (Hymenoptera: Formicidae) on the Greek island of Rhodes**. *Myrmecol News* 2012, **17**:5–11.
10. Katoh K, Asimenos G, Toh H: **Multiple alignment of DNA sequences with MAFFT**. In: *Bioinformatics for DNA sequence analysis.* Springer; 2009: 39–64.
11. Faircloth B: **Illumiprocessor: a trimmomatic wrapper for parallel adapter and quality trimming**. 2013. http://dx.doi.org/10.6079/J9ILL.
12. Bolger AM, Lohse M, Usadel B: **Trimmomatic: a flexible trimmer for Illumina sequence data**. *Bioinformatics* 2014:btu170.
13. Faircloth B: **PHYLUCE is a software package for the analysis of conserved genomic loci**. 2015. http://dx.doi:10.6079/J9PHYL.
14. Crawford NG, Faircloth BC, McCormack JE, Brumfield RT, Winker K, Glenn TC: **More than 1000 ultraconserved elements provide evidence that turtles are the sister group of archosaurs**. *Biol Lett* 2012, **8**:783–786.
15. Grabherr MG, Haas BJ, Yassour M, Levin JZ, Thompson DA, Amit I, Adiconis X, Fan L, Raychowdhury R, Zeng Q *et al*: **Full-length transcriptome assembly from RNA-Seq data without a reference genome**. *Nat Biotech* 2011, **29**:644–652.
16. Castresana J: **Selection of conserved blocks from multiple alignments for their use in phylogenetic analysis**. *Mol Biol Evol* 2000, **17**:540–552.

17. Lanfear R, Calcott B, Ho SYW, Guindon S: **PartitionFinder: Combined selection of partitioning schemes and substitution models for phylogenetic analyses**. *Mol Biol Evol* 2012, **29**:1695–1701.
18. Stamatakis A: **RAxML-VI-HPC: maximum likelihood-based phylogenetic analyses with thousands of taxa and mixed models**. *Bioinformatics* 2006, **22**:2688–2690.
19. Zwickl DJ: **Genetic algorithm approaches for the phylogenetic analysis of large biological sequence datasets under the maximum likelihood criterion**. The University of Texas at Austin; 2006.
20. Ronquist F, Teslenko M, van der Mark P, Ayres DL, Darling A, Höhna S, Larget B, Liu L, Suchard MA, Huelsenbeck JP: **MrBayes 3.2: efficient Bayesian phylogenetic inference and model choice across a large model space**. *Syst Biol* 2012, **61**:539–542.
21. Frandsen PB, Calcott B, Mayer C, Lanfear R: **Automatic selection of partitioning schemes for phylogenetic analyses using iterative k-means clustering of site rates**. *BMC Evol Biol* 2015, **15**:13.
22. Townsend JP: **Profiling phylogenetic informativeness**. *Syst Biol* 2007, **56**:222–231.
23. Faircloth BC, Chang J, Alfaro ME: **TAPIR enables high-throughput estimation and comparison of phylogenetic informativeness using locus-specific substitution models**. 2012. *arXiv preprint arXiv:12021215*.
24. López-Giráldez F, Townsend JP: **PhyDesign: an online application for profiling phylogenetic informativeness**. *BMC Evol Biol* 2011, **11**:152.
25. Gilbert PS, Chang J, Pan C, Sobel EM, Sinsheimer JS, Faircloth BC, Alfaro ME: **Genome-wide ultraconserved elements exhibit higher phylogenetic informativeness than traditional gene markers in percomorph fishes**. *Mol Phylogenet Evol* 2015, **92**:140–146.
26. Drummond AJ, Suchard MA, Xie D, Rambaut A: **Bayesian Phylogenetics with BEAUti and the BEAST 1.7**. *Mol Biol Evol* 2012, **29**:1969–1973.
27. Francoeur A: **Révision taxonomique des espèces néarctiques du groupe fusca, genre *Formica* (Formicidae, Hymenoptera)**. *Mem. Soc. Entomol. Qué* 1973, **3**:1–316.
28. Ree RH, Smith SA: **Maximum likelihood inference of geographic range evolution by dispersal, local extinction, and cladogenesis**. *Syst Biol* 2008, **57**:4–14.
29. Beaulieu JM, Tank DC, Donoghue MJ: **A Southern Hemisphere origin for campanulid angiosperms, with traces of the break-up of Gondwana**. *BMC Evol Biol* 2013, **13**:80.
30. Yu Y, Harris AJ, Blair C, He X: **RASP (Reconstruct Ancestral State in Phylogenies): a tool for historical biogeography**. *Mol Phylogenet Evol* 2015, **87**:46–49.
31. Ward PS, Brady SG, Fisher BL, Schultz TR: **The evolution of myrmicine ants: phylogeny and biogeography of a hyperdiverse ant clade (Hymenoptera: Formicidae)**. *Syst Ent* 2015, **40**:61–81.
