# Supplementary material for: Phylogenomic methods outperform traditional multi-locus approaches in resolving deep evolutionary history: a case study of formicine ants
Source: BMC Evol Biol. 2015 Dec 4;15:271. doi: 10.1186/s12862-015-0552-5 (PMC4670518; doi:10.1186/s12862-015-0552-5)
Supplement: Additional file 2: — Genbank accessions for PCR-amplified sequences included in this study. Table including specimen identifiers and Genbank accession numbers for all study taxa. (PDF 94 kb) [file 12862_2015_552_MOESM2_ESM.pdf]

Additional file 2: Genbank accessions for PCR-amplified sequences included in this study. Accession numbers are provided for sequences from 10 nuclear markers for 90 formicine and outgroup taxa included in this study. Newly generated and accessioned numbers are displayed in bold font.

Taxon	Voucher (CAS)	18S	28S	Wg	AA	LR	F1	F2	ArgK	Top1	Ubx
Acanthoponera minor	CASENT0039772	EF012825	EF012953	EF013661	EF013081	EF013533	EF013209	EF013371	KJ861140	KJ861751	KJ860480
Acropyga acutiventris	CASENT0106009	EF012827	EF012955	EF013663	EF013083	EF013535	EF013212	EF013374	**KT443544**	**KT443624**	**KT443704**
Acropyga CF01	CASENT0008664	**KT443312**	**KT443370**	**KT443256**	**KT443428**	**KT443486**	**KT443144**	**KT443200**	**KT443545**	**KT443625**	**KT443705**
Agraulomyrmex TZ01	CASENT0217051	**KT443313**	**KT443371**	**KT443257**	**KT443429**	**KT443487**	**KT443145**	**KT443201**	**KT443546**	**KT443626**	**KT443706**
Aneuretus simoni	CASENT0007014	EF012833	EF012961	EF013669	EF013089	EF013541	EF013220	EF013382	FJ939840	KJ523645	KJ523562
Anoplolepis custodiens	CASENT0106259	**KT443314**	**KT443372**	**KT443258**	**KT443430**	**KT443488**	**KT443146**	**KT443202**	**KT443547**	**KT443627**	**KT443707**
Anoplolepis gracilipes	CASENT0106057	EF012836	EF012964	EF013672	EF013092	EF013544	EF013223	EF013385	**KT443548**	**KT443628**	**KT443708**
Aphomomyrmex afer	CASENT0217049	**KT443315**	**KT443373**	**KT443259**	**KT443431**	**KT443489**	**KT443147**	**KT443203**	**KT443549**	**KT443629**	**KT443709**
Bajcaridris theryi	CASENT0235255	**KT443316**	**KT443374**	**KT443260**	**KT443432**	**KT443490**	**KT443148**	**KT443204**	**KT443550**	**KT443630**	**KT443710**
Brachymyrmex BR01	CASENT0217326	**KT443317**	**KT443375**	**KT443261**	**KT443433**	**KT443491**	**KT443149**	**KT443205**	**KT443551**	**KT443631**	**KT443711**
Brachymyrmex depilis	CASENT0106038	EF012844	EF012972	EF013680	EF013100	EF013552	EF013233	EF013395	**KT443552**	**KT443632**	**KT443712**
Calomyrmex albertisi	CASENT0106006	EF012845	EF012973	EF013681	EF013101	EF013553	EF013234	EF013396	**KT443553**	**KT443633**	**KT443713**
Calomyrmex laevissimus	CASENT0106150	**KT443318**	**KT443376**	**KT443262**	**KT443434**	**KT443492**	**KT443150**	**KT443206**	**KT443554**	**KT443634**	**KT443714**
Camponotus BCA01	CASENT0106050	EF012846	EF012974	EF013682	EF013102	EF013554	EF013235	EF013397	**KT443555**	**KT443635**	**KT443715**
Camponotus bedoti_cf	CASENT0106261	**KT443319**	**KT443377**	**KT443263**	**KT443435**	**KT443493**	**KT443151**	**KT443207**	**KT443556**	**KT443636**	**KT443716**
Camponotus claviscapus	CASENT0106332	**KT443320**	**KT443378**	**KT443264**	**KT443436**	**KT443494**	**KT443152**	**KT443208**	**KT443557**	**KT443637**	**KT443717**
Camponotus conithorax	CASENT0106008	EF012847	EF012975	EF013683	EF013103	EF013555	EF013236	EF013398	**KT443558**	**KT443638**	**KT443718**
Camponotus gibbinotus	CASENT0106239	**KT443321**	**KT443379**	**KT443265**	**KT443437**	**KT443495**	**KT443153**	**KT443209**	**KT443559**	**KT443639**	**KT443719**
Camponotus gigas	CASENT0106240	**KT443322**	**KT443380**	**KT443266**	**KT443438**	**KT443496**	**KT443154**	**KT443210**	**KT443560**	**KT443640**	**KT443720**
Camponotus hyatti	CASENT0106032	EF012848	EF012976	EF013684	EF013104	EF013556	EF013237	EF013399	**KT443561**	**KT443641**	**KT443721**
Camponotus maritimus	CASENT0106083	AY867448	AY867464	AY867433	AY867480	AY867495	EF013238	EF013400	**KT443562**	**KT443642**	**KT443722**
Camponotus MG001	CASENT0499291	**KT443323**	**KT443381**	**KT443267**	**KT443439**	**KT443497**	**KT443155**	**KT443211**	**KT443563**	**KT443643**	**KT443723**
Camponotus MG089	CASENT0121990	**KT443324**	**KT443382**	**KT443268**	**KT443440**	**KT443498**	**KT443156**	**KT443212**	**KT443564**	**KT443644**	**KT443724**
Camponotus MG131	CASENT0159208	**KT443325**	**KT443383**	**KT443269**	**KT443441**	**KT443499**	**KT443157**	**KT443213**	**KT443565**	**KT443645**	**KT443725**
Camponotus saundersi	CASENT0106127	**KT443326**	**KT443384**	**KT443270**	**KT443442**	**KT443500**	**KT443158**	**KT443214**	**KT443566**	**KT443646**	**KT443726**
Camponotus vitiensis	CASENT0106151	**KT443327**	**KT443385**	**KT443271**	**KT443443**	**KT443501**	**KT443159**	**KT443215**	**KT443567**	**KT443647**	**KT443727**
Cataglyphis cursor	CASENT0106260	**KT443328**	**KT443386**	**KT443272**	**KT443444**	**KT443502**	**KT443160**	**KT443216**	**KT443568**	**KT443648**	**KT443728**
Cladomyrma petalae	CASENT0235222	**KT443329**	**KT443387**	**KT443273**	**KT443445**	**KT443503**	**KT443161**	**KT443217**	**KT443569**	**KT443649**	**KT443729**
Dolichoderus pustulatus	CASENT0106164	FJ939760	FJ939792	FJ940028	FJ939824	FJ939995	FJ940060	FJ939963	FJ939859	KJ523685	KJ523602
Echinopla australis	CASENT0106149	**KT443330**	**KT443388**	**KT443274**	**KT443446**	**KT443504**	**KT443162**	**KT443218**	**KT443570**	**KT443650**	**KT443730**
Echinopla striata_nr	CASENT0106129	**KT443331**	**KT443389**	**KT443275**	**KT443447**	**KT443505**	**KT443163**	**KT443219**	**KT443571**	**KT443651**	**KT443731**
Euprenolepis procera	CASENT0106328	**KT443332**	**KT443390**	**KT443276**	**KT443448**	**KT443506**	**KT443164**	**KT443220**	**KT443572**	**KT443652**	**KT443732**

Forelophilus philippinensis_cf	CASENT0106324	**KT443333**	**KT443391**	**KT443277**	**KT443449**	**KT443507**	**KT443165**	**KT443221**	**KT443573**	**KT443653**	**KT443733**
Formica moki	CASENT0106084	AY703493	AY703560	AY703627	AY703694	AY703761	EF013263	EF013425	**KT443574**	**KT443654**	**KT443734**
Formica neogagates	CASENT0106302	**KT443334**	**KT443392**	**KT443278**	**KT443450**	**KT443508**	**KT443166**	**KT443222**	**KT443575**	**KT443655**	**KT443735**
Gesomyrmex KH01	CASENT0106178	**KT443335**	**KT443393**	**KT443279**	**KT443451**	**KT443509**	**KT443167**	**KT443223**	**KT443576**	**KT443656**	**KT443736**
Gesomyrmex TH01	CASENT0118772	**KT443336**	**KT443394**	**KT443280**	**KT443452**	**KT443510**	**KT443168**	**KT443224**	**KT443577**	**KT443657**	**KT443737**
Gigantiops destructor	CASENT0106169	**KT443337**	**KT443395**	**KT443281**	**KT443453**	**KT443511**	**KT443169**	**KT443225**	**KT443578**	**KT443658**	**KT443738**
Iberoformica subrufa	CASENT0270631	**KT443338**	**KT443396**	**KT443282**	**KT443454**	**KT443512**	**KT443170**	**KT443226**	**KT443579**	**KT443659**	**KT443739**
Lasiophanes atriventris	CASENT0106235	**KT443339**	**KT443397**	**KT443283**	**KT443455**	**KT443513**	**KT443171**	**KT443227**	**KT443580**	**KT443660**	**KT443740**
Lasius californicus	CASENT0106045	EF012870	EF012998	EF013706	EF013126	EF013578	EF013268	EF013430	KJ523346	KJ523692	KJ523609
Lasius niger	CASENT0106128	**KT443340**	**KT443398**	**KT443284**	**KT443456**	**KT443514**	**KT443172**	**KT443228**	**KT443581**	**KT443661**	**KT443741**
Lepisiota AFRC-LIM-03	CASENT0280596	**KT443341**	**KT443399**	**KT443285**	**KT443457**	**KT443515**	**KT443173**	**KT443229**	**KT443582**	**KT443662**	**KT443742**
Lepisiota canescens	CASENT0138305	**KT443342**	**KT443400**	**KT443286**	**KT443458**	**KT443516**	**KT443174**	**KT443230**	**KT443583**	**KT443663**	**KT443743**
Manica bradleyi	CASENT0106022	EF012878	EF013006	EF013714	EF013134	EF013586	EF013281	EF013443	FJ939877	KJ523698	KJ523615
Melophorus AU01	CASENT0106148	**KT443343**	**KT443401**	**KT443287**	**KT443459**	**KT443517**	**KT443175**	**KT443231**	**KT443584**	**KT443664**	**KT443744**
Myrmecia pyriformis	CASENT0106088	AY703500	AY703567	AY703634	AY703701	AY703768	EF013292	EF013454	FJ939878	KJ523699	KJ523616
Myrmecocystus flaviceps	CASENT0106055	EF012888	EF013016	EF013724	EF013144	EF013596	EF013294	EF013456	**KT443585**	**KT443665**	**KT443745**
Myrmecorhynchus emeryi	CASENT0227590	**KT443344**	**KT443402**	**KT443288**	**KT443460**	**KT443518**	**KT443176**	**KT443232**	**KT443586**	**KT443666**	**KT443746**
Myrmelachista flavocotea	CASENT0106049	EF012889	EF013017	EF013725	EF013145	EF013597	EF013295	EF013457	KJ861144	KJ861755	KJ860484
Myrmoteras iriodum	CASENT0006837	EF012893	EF013021	EF013729	EF013149	EF013601	EF013300	EF013462	**KT443587**	**KT443667**	**KT443747**
Nothomyrmecia macrops	CASENT0106089	AY703501	AY703568	AY703635	AY703702	AY703769	EF013304	EF013466	FJ939880	KJ523703	KJ523620
Notoncus capitatus	CASENT0106015	EF012896	EF013024	EF013732	EF013152	EF013604	EF013305	EF013467	**KT443588**	**KT443668**	**KT443748**
Notostigma carazzii	CASENT0106112	EF012897	EF013025	EF013733	EF013153	EF013605	EF013306	EF013468	**KT443589**	**KT443669**	**KT443749**
Nylanderia dodo	CASENT0059506	**KT443345**	**KT443403**	FJ982532	**KT443461**	**KT443519**	FJ982494	FJ982574	**KT443590**	**KT443670**	**KT443750**
Nylanderia hystrix	CASENT0106037	EF012906	EF013034	EF013742	EF013162	EF013614	EF013316	EF013478	**KT443591**	**KT443671**	**KT443751**
Nylanderia MG01	CASENT0049168	**KT443346**	**KT443404**	FJ982540	**KT443462**	**KT443520**	FJ982502	FJ982582	**KT443592**	**KT443672**	**KT443752**
Oecophylla longinoda	CASENT0106331	**KT443347**	**KT443405**	**KT443289**	**KT443463**	**KT443521**	**KT443177**	**KT443233**	**KT443593**	**KT443673**	**KT443753**
Oecophylla smaragdina	CASENT0106113	EF012900	EF013028	EF013736	EF013156	EF013608	EF013309	EF013471	**KT443594**	**KT443674**	**KT443754**
Opisthopsis PG01	CASENT0106170	**KT443348**	**KT443406**	**KT443290**	**KT443464**	**KT443522**	**KT443178**	**KT443234**	**KT443595**	**KT443675**	**KT443755**
Opisthopsis respiciens	CASENT0106020	EF012902	EF013030	EF013738	EF013158	EF013610	EF013311	EF013473	**KT443596**	**KT443676**	**KT443756**
Paraparatrechina glabra	CASENT0067057	**KT443349**	**KT443407**	**KT443291**	**KT443465**	**KT443523**	**KT443179**	**KT443235**	**KT443597**	**KT443677**	**KT443757**
Paraparatrechina oceanica	CASENT0106330	**KT443350**	**KT443408**	**KT443292**	**KT443466**	**KT443524**	**KT443180**	**KT443236**	**KT443598**	**KT443678**	**KT443758**
Paratrechina antsingy	CASENT0914212	**KT443351**	**KT443409**	**KT443293**	**KT443467**	**KT443525**	**KT443181**	**KT443237**	**KT443599**	**KT443679**	**KT443759**
Paratrechina longicornis	CASENT0106255	**KT443352**	**KT443410**	**KT443294**	**KT443468**	**KT443526**	**KT443182**	**KT443238**	**KT443600**	**KT443680**	**KT443760**
Paratrechina zanjensis	CASENT0280583	**KT443353**	**KT443411**	**KT443295**	**KT443469**	**KT443527**	**KT443183**	**KT443239**	**KT443601**	**KT443681**	**KT443761**
Petalomyrmex phylax	CASENT0217048	**KT443354**	**KT443412**	**KT443296**	**KT443470**	**KT443528**	**KT443184**	**KT443240**	**KT443602**	**KT443682**	**KT443762**
Phasmomyrmex ZA01	CASENT0217047	**KT443355**	**KT443413**	**KT443297**	**KT443471**	**KT443529**	**KT443185**	**KT443241**	**KT443603**	**KT443683**	**KT443763**
Plagiolepis alluaudi	CASENT0077847	**KT443356**	**KT443414**	**KT443298**	**KT443472**	**KT443530**	**KT443186**	**KT443242**	**KT443604**	**KT443684**	**KT443764**

Plagiolepis MG05	CASENT0107071	**KT443357**	**KT443415**	**KT443299**	**KT443473**	**KT443531**	**KT443187**	**KT443243**	**KT443605**	**KT443685**	**KT443765**
Polyergus breviceps	CASENT0106048	EF012915	EF013043	EF013751	EF013171	EF013623	EF013326	EF013488	**KT443606**	**KT443686**	**KT443766**
Polyrhachis decumbens	CASENT0106014	EF012916	EF013044	EF013752	EF013172	EF013624	EF013327	EF013489	**KT443607**	**KT443687**	**KT443767**
Polyrhachis Hagio01	CASENT0106007	EF012917	EF013045	EF013753	EF013173	EF013625	EF013328	EF013490	**KT443608**	**KT443688**	**KT443768**
Prenolepis emmae	CASENT0106329	**KT443358**	**KT443416**	**KT443300**	**KT443474**	**KT443532**	**KT443188**	**KT443244**	**KT443609**	**KT443689**	**KT443769**
Prenolepis imparis	CASENT0106035	EF012919	EF013047	EF013755	EF013175	EF013627	EF013330	EF013492	**KT443610**	**KT443690**	**KT443770**
Proformica mongolica	CASENT0106258	**KT443359**	**KT443417**	**KT443301**	**KT443475**	**KT443533**	**KT443189**	**KT443245**	**KT443611**	**KT443691**	**KT443771**
Prolasius convexus	CASENT0227589	**KT443360**	**KT443418**	**KT443302**	**KT443476**	**KT443534**	**KT443190**	**KT443246**	**KT443612**	**KT443692**	**KT443772**
Pseudolasius australis	CASENT0106005	EF012927	EF013055	EF013763	EF013183	EF013635	EF013339	EF013501	**KT443613**	**KT443693**	**KT443773**
Pseudonotoncus hirsutus	CASENT0227592	**KT443361**	**KT443419**	**KT443303**	**KT443477**	**KT443535**	**KT443191**	**KT443247**	**KT443614**	**KT443694**	**KT443774**
Rhytidoponera chalybaea	CASENT0106000	EF012930	EF013058	EF013766	EF013186	EF013638	EF013343	EF013505	FJ939885	KJ523705	KJ523622
Rossomyrmex anatolicus	CASENT0235254	**KT443362**	**KT443420**	**KT443304**	**KT443478**	**KT443536**	**KT443192**	**KT443248**	**KT443615**	**KT443695**	**KT443775**
Santschiella kohli	CASENT0004701	**KT443363**	**KT443421**	**KT443305**	**KT443479**	**KT443537**	**KT443193**	**KT443249**	**KT443616**	**KT443696**	**KT443776**
Stigmacros clivispina_cf	CASENT0106256	**KT443364**	**KT443422**	**KT443306**	**KT443480**	**KT443538**	**KT443194**	**KT443250**	**KT443617**	**KT443697**	**KT443777**
Tapinolepis MG01	CASENT0041468	**KT443365**	**KT443423**	**KT443307**	**KT443481**	**KT443539**	**KT443195**	**KT443251**	**KT443618**	**KT443698**	**KT443778**
Tapinolepis ZA01	CASENT0217038	**KT443366**	**KT443424**	**KT443308**	**KT443482**	**KT443540**	**KT443196**	**KT443252**	**KT443619**	**KT443699**	**KT443779**
Formicinae_genus_01 ZA02	CASENT0217131	**KT443367**	**KT443425**	**KT443309**	**KT443483**	**KT443541**	**KT443197**	**KT443253**	**KT443620**	**KT443700**	**KT443780**
Formicinae_genus_01 ZA03	CASENT0217132	**KT443368**	**KT443426**	**KT443310**	**KT443484**	**KT443542**	**KT443198**	**KT443254**	**KT443621**	**KT443701**	**KT443781**
Teratomyrmex greavesi	QMT162820	**KT443369**	**KT443427**	**KT443311**	**KT443485**	**KT443543**	**KT443199**	**KT443255**	**KT443622**	**KT443702**	**KT443782**
Tetraponera rufonigra	CASENT0106099	AY703515	AY703582	AY703649	AY703716	AY703783	EF013362	EF013524	FJ939892	KJ523718	KJ523635
Zatania albimaculata	CASENT0106052	EF012918	EF013046	EF013754	EF013174	EF013626	EF013329	EF013491	**KT443623**	**KT443703**	**KT443783**
