# Supplementary material for: Phylogenomic methods outperform traditional multi-locus approaches in resolving deep evolutionary history: a case study of formicine ants
Source: BMC Evol Biol. 2015 Dec 4;15:271. doi: 10.1186/s12862-015-0552-5 (PMC4670518; doi:10.1186/s12862-015-0552-5)
Supplement: Additional file 3: — Calibration points used to define prior calibration densities for dating analyses with BEAST. (PDF 91 kb) [file 12862_2015_552_MOESM3_ESM.pdf]

Additional file 3: Calibration points used for dating analyses with BEAST.

Taxon (stem-group)	Distribution	5%	Offset	Median	95%	Fossil and Formation
Acropyga	lognormal		15	40	80	1 species of *Acropyga* in Dominican amber [1].
Camponotus (s.l.)	lognormal		42	60	90	1 species of *Camponotus* in Baltic amber [2].
Formica	lognormal		42	60	90	*Formica* in Baltic amber [2].
Formicinae	lognormal		92	95	120	*Kyromyrma* in New Jersey amber [3].
Lasius	lognormal		42	60	90	9 species of *Lasius* in Baltic and other late Eocene ambers [2].
Plagiolepis	lognormal		42	55	80	6 species of *Plagiolepis* Baltic and other late Eocene ambers [4].
Prenolepis (*P. imparis*)	lognormal		42	55	80	*Prenolepis* in Baltic amber [2].
Pseudolasius	lognormal		42	55	80	*Pseudolasius* in Baltic amber [5].
Root	normal	100			130	95% HPD [100,130] as estimated in Brady et al. [6].

References

1. LaPolla JS: **Ancient trophophoresy: a fossil *Acropyga* (Hymenoptera: Formicidae) from Dominican amber**. *Trans Am Entomol Soc* 2005:21–28.
2. Dlussky GM: **Genera of ants (Hymenoptera: Formicidae) from Baltic amber**. *Paleontol J* 1997, **31**:616–627.
3. Grimaldi D, Agosti D: **A formicine in New Jersey Cretaceous amber (Hymenoptera: Formicidae) and early evolution of the ants**. *Proc Natl Acad Sci* 2000, **97**:13678–13683.
4. Dlussky GM: **Ants of the genus *Plagiolepis* Mayr (Hymenoptera, Formicidae) from Late Eocene ambers of Europe**. *Paleontol J* 2010, **44**:546–555.
5. LaPolla JS, Dlussky GM: **Review of fossil *Prenolepis* genus-group species (Hymenoptera: Formicidae)**. *Proc Entomol Soc Wash* 2010, **112**:258–273.
6. Brady SG, Fisher BL, Schultz TR, Ward PS: **The rise of army ants and their relatives: diversification of specialized predatory doryline ants**. *BMC Evol Biol* 2014, **14**:93.
