# Supplementary material for: Phylogenomic methods outperform traditional multi-locus approaches in resolving deep evolutionary history: a case study of formicine ants
Source: BMC Evol Biol. 2015 Dec 4;15:271. doi: 10.1186/s12862-015-0552-5 (PMC4670518; doi:10.1186/s12862-015-0552-5)
Supplement: Additional file 4: — Taxon distribution matrix used in the biogeographic analyses. (PDF 81 kb) [file 12862_2015_552_MOESM4_ESM.pdf]

Additional file 4: Taxon distribution matrix used in the biogeographic analyses. T = Neotropical, N = Neartic, P = Palearctic, E = Afrotropical, O = Indomalayan, A = Australasian.

**Species**	**T**	**N**	**P**	**E**	**O**	**A**	**Species**	**T**	**N**	**P**	**E**	**O**	**A**
Acropyga_acutiventris	1	1	1	1	1	1	*Lepisiota*_AFRC-LIM-03	0	0	0	1	0	0
*Acropyga*_CF01	0	0	0	1	0	0	*Lepisiota_canescens*	0	0	1	1	1	0
*Agraulomyrmex*_TZ01	0	0	0	1	0	0	*Melophorus*_AU01	0	0	0	0	0	1
Anoplolepis_custodiens	0	0	0	1	0	0	*Myrmecocystus_flaviceps*	0	1	0	0	0	0
Anoplolepis_gracilipes	0	0	0	0	1	0	*Myrmecorhynchus_emeryi*	0	0	0	0	0	1
Aphomomyrmex_afer	0	0	0	1	0	0	*Myrmelachista_flavocotea*	1	0	0	0	0	0
Bajcaridris_theryi	0	0	1	0	0	0	*Myrmoteras_iriodum*	0	0	0	0	1	0
Brachymyrmex_BR01	1	0	0	0	0	0	*Notoncus_capitatus*	0	0	0	0	0	1
Brachymyrmex_depilis	1	1	0	0	0	0	*Notostigma_carazzii*	0	0	0	0	0	1
Calomyrmex_albertisi	0	0	0	0	0	1	*Nylanderia_dodo*	1	0	0	1	1	1
Calomyrmex_laevissimus	0	0	0	0	0	1	*Nylanderia_hystrix*	1	1	0	0	0	0
*Camponotus*_BCA01	1	1	1	0	0	0	*Nylanderia_MG01*	1	0	0	1	1	1
Camponotus_bedoti_cf	0	0	0	0	1	1	*Oecophylla_longinoda*	0	0	0	1	0	0
Camponotus_claviscapus	1	0	0	0	0	0	*Oecophylla_smaragdina*	0	0	0	0	1	1
Camponotus_conithorax	0	0	0	0	1	1	*Opisthopsis_PG01*	0	0	0	0	0	1
Camponotus_gibbinotus	0	0	0	0	1	1	*Opisthopsis_respiciens*	0	0	0	0	0	1
Camponotus_hyatti	1	1	1	0	0	0	*Paraparatrechina_glabra*	0	0	0	1	0	0
Camponotus_maritimus	1	1	1	0	0	0	*Paraparatrechina_oceanica*	0	0	0	0	1	1
*Camponotus*_MG001	0	0	0	1	0	0	*Paratrechina_antsingy*	0	0	0	1	0	0
*Camponotus*_MG089	0	0	0	1	0	0	*Paratrechina_longicornis*	0	0	0	1	0	0
*Camponotus*_MG131	0	0	0	1	0	0	*Paratrechina_zanjensis*	0	0	0	1	0	0
Camponotus_saundersi	0	0	0	0	1	0	*Petalomyrmex_phylax*	0	0	0	1	0	0
Camponotus_vitiensis	0	0	0	0	0	1	*Phasmomyrmex*_ZA01	0	0	0	1	0	0
Cataglyphis_cursor	0	0	1	1	1	0	*Plagiolepis_alluaudi*	0	0	1	1	1	1
Cladomyrma_petalae	0	0	0	0	1	0	*Plagiolepis*_MG05	0	0	0	1	0	0
Camponotus_gigas	0	0	0	0	1	0	*Polyergus_breviceps*	0	1	1	0	0	0
Echinopla_australis	0	0	0	0	1	1	*Polyrhachis_decumbens*	0	0	0	0	1	1
Echinopla_striata_nr	0	0	0	0	1	1	*Polyrhachis*_Hagio01	0	0	0	1	1	1
Euprenolepis_procera	0	0	0	0	1	0	*Prenolepis_emmae*	0	0	0	0	1	0
Forelophilus_philippinensis_cf	0	0	0	0	1	0	*Prenolepis_imparis*	0	1	1	1	1	0
Formica_moki	1	1	1	0	0	0	*Proformica_mongolica*	0	0	1	0	0	0
Formica_neogagates	0	1	0	0	0	0	*Prolasius_convexus*	0	0	0	0	0	1
*Formicine_genus_01*_ZA02	0	0	0	1	0	0	*Pseudolasius_australis*	0	0	0	1	1	1
*Formicine_genus-01*_ZA03	0	0	0	1	0	0	*Pseudonotoncus_hirsutus*	0	0	0	0	0	1
*Gesomyrmex*_KH01	0	0	0	0	1	0	*Rossomyrmex_anatolicus*	0	0	1	0	0	0
*Gesomyrmex*_TH01	0	0	0	0	1	0	*Santschiella_kohli*	0	0	0	1	0	0
Gigantiops_destructor	1	0	0	0	0	0	*Stigmacros_clivispina_cf*	0	0	0	0	0	1
Iberoformica_subrufa	0	0	1	0	0	0	*Tapinolepis*_MG01	0	0	0	1	0	0
Lasiophanes_atriventris	1	0	0	0	0	0	*Tapinolepis*_ZA01	0	0	0	1	0	0
Lasius_californicus	0	1	0	0	0	0	*Teratomyrmex_greavesi*	0	0	0	0	0	1
Lasius_niger	0	1	1	0	0	0	*Zatania_albimaculata*	1	0	0	0	0	0
