# Supplementary material for: Phylogenomic methods outperform traditional multi-locus approaches in resolving deep evolutionary history: a case study of formicine ants
Source: BMC Evol Biol. 2015 Dec 4;15:271. doi: 10.1186/s12862-015-0552-5 (PMC4670518; doi:10.1186/s12862-015-0552-5)
Supplement: Additional file 5: — Dispersal constraint matrices used for DEC and S-DEC analyses in RASP. (PDF 75 kb) [file 12862_2015_552_MOESM5_ESM.pdf]

**Additional file 5: Dispersal constraint matrices used for DEC and S-DEC analyses.** Probabilities were assigned similarly to Ward et al. (2015): 0.1 to adjacent areas, 0.5 to adjacent areas separated by a large body of water, and 0.01 to geographically disjunct areas. T=Neotropical, N=Neartic, P=Palearctic, E=Afrotropical, O=Indomalayan, A=Australasian. The two time slices represent changing connectivity between these regions in accordance with paleogeographic estimates (Scotese 2010).

| **0-50 Ma** | **T** | **N** | **P** | **E** | **O** | **A** |
|---|---|---|---|---|---|---|
| **T** | / | 1.0 | 0.01 | 0.5 | 0.01 | 0.5 |
| **N** | 1.0 | / | 1.0 | 0.01 | 0.01 | 0.01 |
| **P** | 0.01 | 1.0 | / | 1.0 | 1.0 | 0.01 |
| **E** | 0.5 | 0.01 | 1.0 | / | 1.0 | 0.5 |
| **O** | 0.01 | 0.01 | 1.0 | 1.0 | / | 1.0 |
| **A** | 0.5 | 0.01 | 0.01 | 0.5 | 1.0 | / |
| **50-105 Ma** | **T** | **N** | **P** | **E** | **O** | **A** |
| **T** | / | 1.0 | 0.01 | 1.0 | 0.01 | 1.0 |
| **N** | 1.0 | / | 1.0 | 0.01 | 0.01 | 0.01 |
| **P** | 0.01 | 1.0 | / | 1.0 | 1.0 | 0.01 |
| **E** | 1.0 | 0.01 | 1.0 | / | 1.0 | 1.0 |
| **O** | 0.01 | 0.01 | 1.0 | 1.0 | / | 0.5 |
| **A** | 1.0 | 0.01 | 0.01 | 1.0 | 0.5 | / |
