# Supplementary material for: Phylogenomic methods outperform traditional multi-locus approaches in resolving deep evolutionary history: a case study of formicine ants
Source: BMC Evol Biol. 2015 Dec 4;15:271. doi: 10.1186/s12862-015-0552-5 (PMC4670518; doi:10.1186/s12862-015-0552-5)
Supplement: Additional file 6: — Summary of UCE capture statistics. (PDF 95 kb) [file 12862_2015_552_MOESM6_ESM.pdf]

Additional file 6: Summary of UCE capture statistics.

Table listing the total UCE read count and total average read length; the total number of contigs recovered, their coverage (X) and mean length; the number of assembled UCE contigs and their mean length and mean coverage for each taxon included in the analyses.

Taxon	Total read count	Total average read length	Total contigs (after trimming)	Total contigs coverage (x)	Total contigs mean length	UCE contigs	UCE contigs mean length	UCE contigs mean coverage
Acanthoponera minor	2,135,179	275	46732	13.1	303.8	1032	884.6	119.89
Acropyga acutiventris	3,072,893	295	32768	20	337.1	908	1057.2	198.2
Acropyga CF01	852,471	257	13422	16.9	297.6	1014	529.1	66.5
Agraulomyrmex TZ01	359,942	291	4383	19	357.4	851	479.8	35.8
Aneuretus simoni	2,097,248	274	46245	12.9	293.2	1041	830.1	149.25
Anoplolepis custodiens	1,204,481	278	9853	28.1	449	959	760.5	54.89
Anoplolepis gracilipes	764,452	270	7897	18.5	370.2	918	804.3	57.14
Aphomomyrmex afer	3,073,391	286	53865	18.2	324	1029	825.6	180.10
Bajcaridris theryi	1,271,359	291	10874	30.8	351.7	618	546.9	46.1
Brachymyrmex BR01	148,054	275	2031	17.3	282.4	677	309.6	23
Brachymyrmex depilis	955,852	274	12043	17.8	372.7	967	724.8	50.36
Calomyrmex albertisi	797,286	411	5712	24.1	682.7	970	826.9	47.65
Calomyrmex laevissimus	2,201,212	289	41541	12.1	325.6	1033	1010.3	101
Camponotus BCA01	1,530,707	290	17905	17.9	428.9	919	893.7	79.81
Camponotus bedoti cf	1,807,509	277	29881	14.5	318.4	995	856.2	103.75
Camponotus claviscapus	1,697,038	286	18763	16	420.1	1001	940.1	88
Camponotus conithorax	1,374,110	287	17062	14.3	448.7	1007	921.1	65.75
Camponotus gibbinotus	1,799,933	279	37301	13.1	314.4	981	828.7	123.33
Camponotus hyatti	2,168,078	279	44013	12.8	306.7	961	891.3	105.94
Camponotus maritimus	1,024,917	302	14331	15.6	468.8	943	847.6	70.40
Camponotus MG001	912,128	273	12061	16.4	360	899	846.9	75.03
Camponotus MG089	2,851,670	296	90482	11	305.7	997	922.5	180.93
Camponotus MG131	875,269	273	9130	19.7	387.2	950	849.6	62.97
Camponotus saundersi	951,409	271	9932	15.2	373.9	929	897.3	52.74
Camponotus vitiensis	840,889	274	6756	21.8	439.8	933	868.4	45.55
Cataglyphis cursor	1,557,357	271	20147	16.1	325.6	1000	815.2	84.94
Cladomyrma petalae	2,514,799	301	45768	12	351.6	985	1003.5	116
Dinomyrmex gigas	1,447,691	278	17971	20.2	371.7	941	847.1	81.23
Dolichoderus pustulatus	1,590,104	276	31071	11.8	315	1030	877.8	78.99
Echinopla australis	684,056	281	5975	19.7	452.6	935	815.4	34.14
Echinopla striata nr	1,193,066	273	13639	18.5	370.7	949	859.5	52.76
Euprenolepis procera	1,297,313	272	13040	19.4	376.2	983	925.6	77.1

**Taxon**	**Total read count**	**Total average read length**	**Total contigs (after trimming)**	**Total contigs coverage (x)**	**Total contigs mean length**	**UCE contigs**	**UCE contigs mean length**	**UCE contigs mean coverage**
Forelophilus philippinensis	1,870,416	289	20337	17.1	436	1013	894	96.2
Formica moki	2,958,364	293	77142	12.7	305.5	996	925.2	167.17
Formica neogagates	1,530,003	271	26707	14	317.8	1005	878.9	82.69
Formicine_genus_01_ZA02	1,077,246	268	18412	13.2	322.5	985	792.2	59.52
Formicine_genus_01_ZA03	2,763,024	289	85494	11.2	295.4	990	900.8	160.65
Gesomyrmex KH01	1,304,640	266	25743	15.5	304	935	772.1	84.74
Gesomyrmex TH01	1,514,480	323	24071	19.5	354.7	467	515.2	49.9
Gigantiops destructor	2,949,839	287	54735	14.3	311	1012	804	123.53
Iberoformica subrufa	2,261,168	285	29743	18.2	354.7	1034	983.5	109.9
Lasiophanes atriventris	2,563,885	284	62738	13.1	291.8	1072	799.4	168.87
Lasius californicus	1,432,247	275	27370	12.4	317.7	963	870.8	83.11
Lasius niger	379,238	294	4127	16.5	449.8	873	744.2	28.66
Lepisiota AFRC-LIM-03	1,450,179	314	21393	20.2	370.8	718	600.2	58.3
Lepisiota canescens	1,772,200	287	31051	14.6	375.3	987	848.5	83.11
Manica bradleyi	2,406,461	278	43052	14.6	304.7	1035	888.5	107.94
Melophorus AU01	426,908	284	5176	18.6	408.4	854	737.6	32.50
Myrmecia pyriformis	3,192,220	362	57854	12.8	423.7	891	1231	109.5
Myrmecocystus flaviceps	997,124	268	12137	16.7	325.2	975	846.2	79.88
Myrmecorhynchus emeryi	1,046,862	280	13415	15.8	387.4	953	829.6	59.22
Myrmelachista flavocotea	1,767,714	280	21555	17.8	392.1	949	799.8	77.37
Myrmoteras iriodum	2,227,662	278	68744	12.3	288.7	791	628	125.78
Nothomyrmecia macrops	3,013,789	428	57394	13.2	482.4	577	900.1	122.6
Notoncus capitatus	1,896,661	273	34786	14.2	304.3	1007	874.7	102.19
Notostigma carazzii	616,003	259	7734	30.1	306.2	954	521.7	89.86
Nylanderia dodo	3,155,631	290	97412	12	299.4	997	878.8	191.58
Nylanderia hystrix	869,609	266	9570	19.7	359	987	784.8	48.47
Nylanderia MG01	1,280,811	271	24312	14	305.7	867	819.9	86.40
Oecophylla longinoda	925,073	261	8882	20.5	330.5	973	763.6	74.4
Oecophylla smaragdina	2,572,016	285	59492	13.1	289.2	1015	816	168.66
Opisthopsis PG01	1,688,361	267	29981	14.4	296.4	1019	550.6	117.74
Opisthopsis respiciens	1,780,565	278	31514	13.2	326	1020	948.3	106.01
Paraparatrechina glabra	3,978,461	303	134097	12	307.9	1016	856.1	243.34
Paraparatrechina oceanica	1,748,613	275	27897	14.1	317	1029	882.8	114.7
Paratrechina antsingy	1,917,784	277	32140	13.5	306.7	1029	925.1	125.7
Paratrechina longicornis	948,856	266	9699	19.2	335.5	935	773.2	73.68
Paratrechina zanjensis	146,816	307	1633	21.3	368	763	382.6	20.4
Petalomyrmex phylax	2,712,236	280	71844	12.7	279.2	1023	806.4	206.20

Taxon	Total read count	Total average read length	Total contigs (after trimming)	Total contigs coverage (x)	Total contigs mean length	UCE contigs	UCE contigs mean length	UCE contigs mean coverage
Phasmomyrmex ZA01	2,747,933	286	80445	10.9	294.8	994	951.9	138.02
Plagiolepis alluaudi	925,485	265	11233	15.3	345.8	973	829.8	58.12
Plagiolepis MG05	3,784,056	312	126442	11.9	325.7	1005	909.1	251.17
Polyergus breviceps	1,125,146	272	13637	18.4	346.5	968	851.1	75.94
Polyrhachis decumbens	453,338	765	3321	21.9	749.9	916	842.6	35.95
Polyrhachis Hagio01	1,053,814	325	7697	22.5	590.4	964	899.7	64.22
Prenolepis emmae	1,953,604	281	26102	19.5	349.9	994	930.4	100.7
Prenolepis imparis	1,192,038	276	15426	15.6	378.5	1000	880.8	64.97
Proformica mongolica	100,862	234	185	53.6	316.5	72	246.8	9.8
Prolasius convexus	834,816	272	9160	19	380.9	956	726.2	43.49
Pseudolasius australis	1,690,462	287	17713	17.8	448	970	927.4	78.8
Pseudonotoncus hirsutus	1,015,580	314	12308	24.9	378.4	608	607.1	50
Rhytidoponera chalybaea	959,911	277	11162	20	401.4	1007	844.1	56.79
Rossomyrmex anatolicus	1,340,785	261	8919	47.7	332.1	971	650	53.47
Santschiella kohli	623,316	254	5813	25.7	296.9	899	445.1	61.4
Stigmacros clivispina cf	1,293,246	285	17811	15.1	380.6	978	789.4	72.70
Tapinolepis MG01	1,653,292	276	32870	12.6	319.6	1045	871	99.22
Tapinolepis ZA01	2,494,553	290	52617	12.8	322	1040	946.8	114.2
Teratomyrmex greavesi	1,003,197	271	12786	20.8	324.3	830	564.1	74.5
Tetraponera rufonigra	2,692,704	290	69421	12.5	298.2	1017	796.9	160.44
Zatania albimaculata	1,414,555	272	21860	14.8	317.1	971	890.9	92.06
**Total average**	**1,606,086**	**290.1**	**29654.5**	**17.4**	**359.2**	**936.0**	**805.3**	**92.3**
