# Supplementary material for: Phylogenomic methods outperform traditional multi-locus approaches in resolving deep evolutionary history: a case study of formicine ants
Source: BMC Evol Biol. 2015 Dec 4;15:271. doi: 10.1186/s12862-015-0552-5 (PMC4670518; doi:10.1186/s12862-015-0552-5)
Supplement: Additional file 7: — Revised tribal classification for the Formicinae. (PDF 84 kb) [file 12862_2015_552_MOESM7_ESM.pdf]

Additional file 7: Revised tribal classification for the Formicinae

Part 1. List of tribes and included genera.

The tribal classification of Formicinae is here modified to achieve consistency with our molecular phylogenetic results. We maintain the existing classification as far as possible, while striving to ensure that all recognized tribes are monophyletic. Genera known only from fossils are signified with a dagger; most of these are unplaced to tribe and are treated as *incertae sedis* within the subfamily. A formal taxonomic treatment with these proposed changes will appear elsewhere (Ward *et al.*, submitted).

Tribe **Camponotini**
Genera: *Calomyrmex*, *Camponotus*, †*Chimaeromyrma*, *Colobopsis, Dinomyrmex, Echinopla*, *Opisthopsis*, *Overbeckia*, *Polyrhachis*, †*Pseudocamponotus*.

Tribe **Formicini**
Genera: *Alloformica*, *Bajcaridris*, *Cataglyphis*, †*Cataglyphoides*, †*Conoformica*, *Formica*, *Iberoformica*, *Polyergus*, *Proformica*, †*Protoformica*, *Rossomyrmex.*

Tribe **Gesomyrmecini**
Genera: *Gesomyrmex*, †*Prodimorphomyrmex*, †*Sicilomyrmex*.

Tribe **Gigantiopini**
Genus: *Gigantiops*.

Tribe **Lasiini**
Genera: *Euprenolepis*, †*Glaphyromyrmex*, *Lasius*, *Myrmecocystus*, *Nylanderia*, *Paraparatrechina*, *Paratrechina*, *Prenolepis*, *Pseudolasius*, *Zatania*.

Tribe **Melophorini**
Genera: *Lasiophanes*, *Melophorus*, *Myrmecorhynchus*, *Notoncus*, *Notostigma*, *Prolasius*, *Pseudonotoncus*, *Stigmacros*, *Teratomyrmex*.

Tribe **Myrmelachistini**
Genera: *Brachymyrmex*, *Myrmelachista*

Tribe **Myrmoteratini**
Genus: *Myrmoteras*.

Tribe **Oecophyllini**
Genus: *Oecophylla*.

Tribe **Plagiolepidini**
Genera: *Acropyga*, *Agraulomyrmex*, *Anoplolepis*, *Aphomomyrmex*, *Bregmatomyrma*, *Cladomyrma*, *Lepisiota*, *Petalomyrmex*, *Plagiolepis*, *Tapinolepis*.

Tribe **Santschiellini**
Genus: Santschiella

Unplaced to tribe
Genera: †*Camponotites*, †*Curtipalpulus*, †*Drymomyrmex*, †*Eoleptocerites*, †*Eurytarsites*, †*Fushuniformica*, †*Heeridris*, †*Huaxiaformica*, †*Imhoffia*, †*Kyromyrma*, †*Leptogasteritus*, †*Leucotaphus*, †*Liaoformica*, †*Longiformica*, †*Magnogasterites*, †*Orbicapitia*, †*Ovalicapito*, †*Ovaligastrula*, †*Protrechina*, †*Sinoformica*, †*Sinotenuicapito*, †*Wilsonia*.

Part 2. Generic changes in tribe Camponitini

In an attempt to restore monophyly of the large, cosmopolitan genus *Camponotus* the following generic changes are proposed (Ward *et al.*, submitted): (1) *Colobopsis*, formerly a subgenus of *Camponotus*, is resurrected as a genus; (2) *Dinomyrmex*, formerly a subgenus of *Camponotus*, is resurrected as a genus; (3) *Forelophilus* is synonymized under *Camponotus*; and (4) *Phasmomyrmex* is synonymized under *Camponotus*.

Reference

Ward PS, Fisher BL, Blaimer BB: A revised phylogenetic classification of the ant subfamily Formicinae (Hymenoptera: Formicidae), with resurrection of the genera *Colobopsis* and *Dinomyrmex*. *Zootaxa*, submitted.
