# Supplementary material for: Phylogenomic methods outperform traditional multi-locus approaches in resolving deep evolutionary history: a case study of formicine ants
Source: BMC Evol Biol. 2015 Dec 4;15:271. doi: 10.1186/s12862-015-0552-5 (PMC4670518; doi:10.1186/s12862-015-0552-5)
Supplement: Additional file 8: — Phylogenetic trees from analyses not illustrated in the main text. Additional results from Bayesian and Maximum Likelihood bootstrap analyses. (PDF 2225 kb) [file 12862_2015_552_MOESM8_ESM.pdf]

Additional file 8: Phylogenetic trees from analyses not illustrated in the main text.
A) RAxML bootstrap tree for UCE-70% data set; B) RAxML bootstrap tree for 10-gene data set; C) Bayesian consensus for 10-gene data. RAxML estimations comprised 100 (UCE) and 1152 (10-gene) bootstrap replicates; Bayesian tree summarizes two independent runs of 40 million generations each.

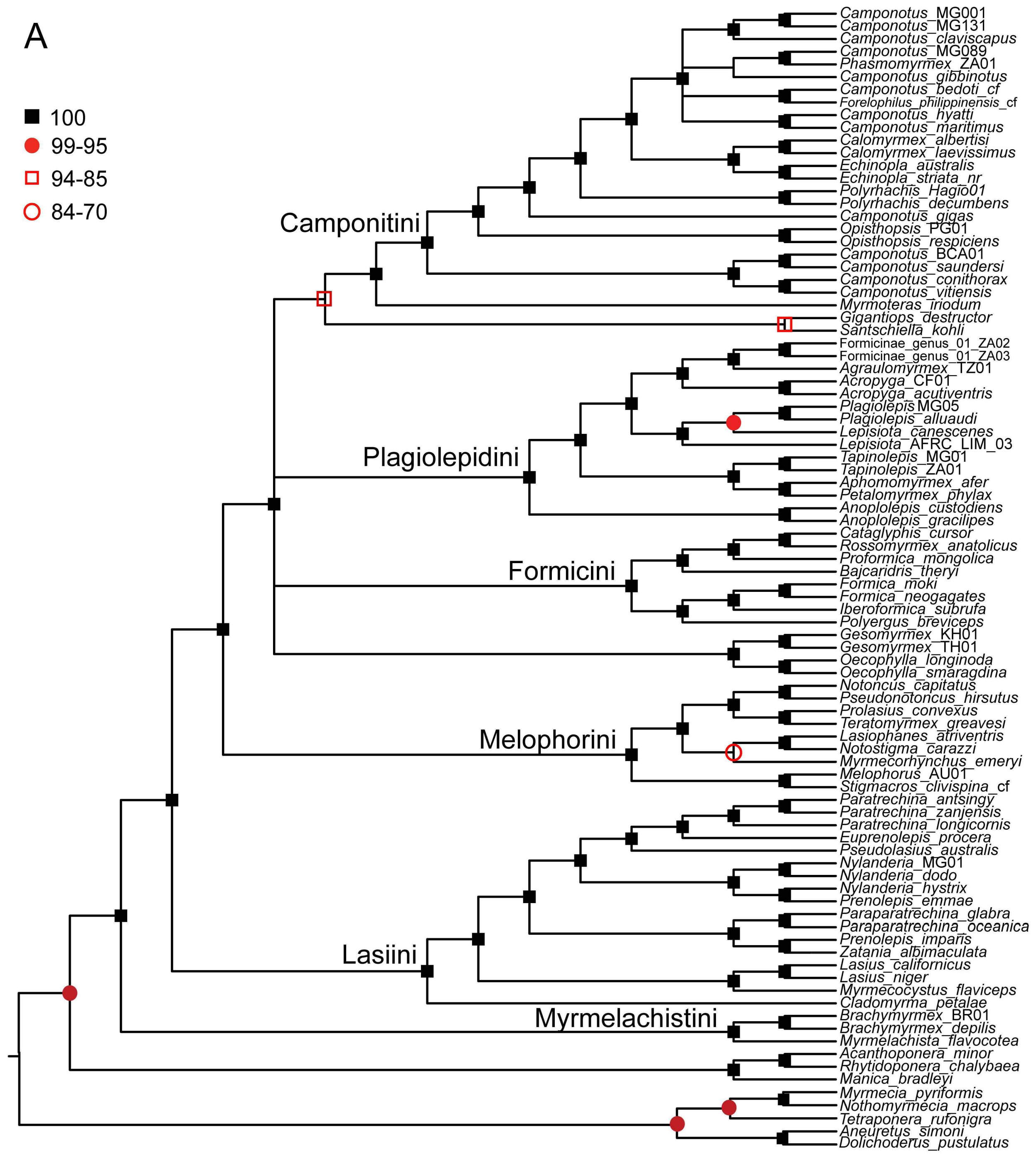

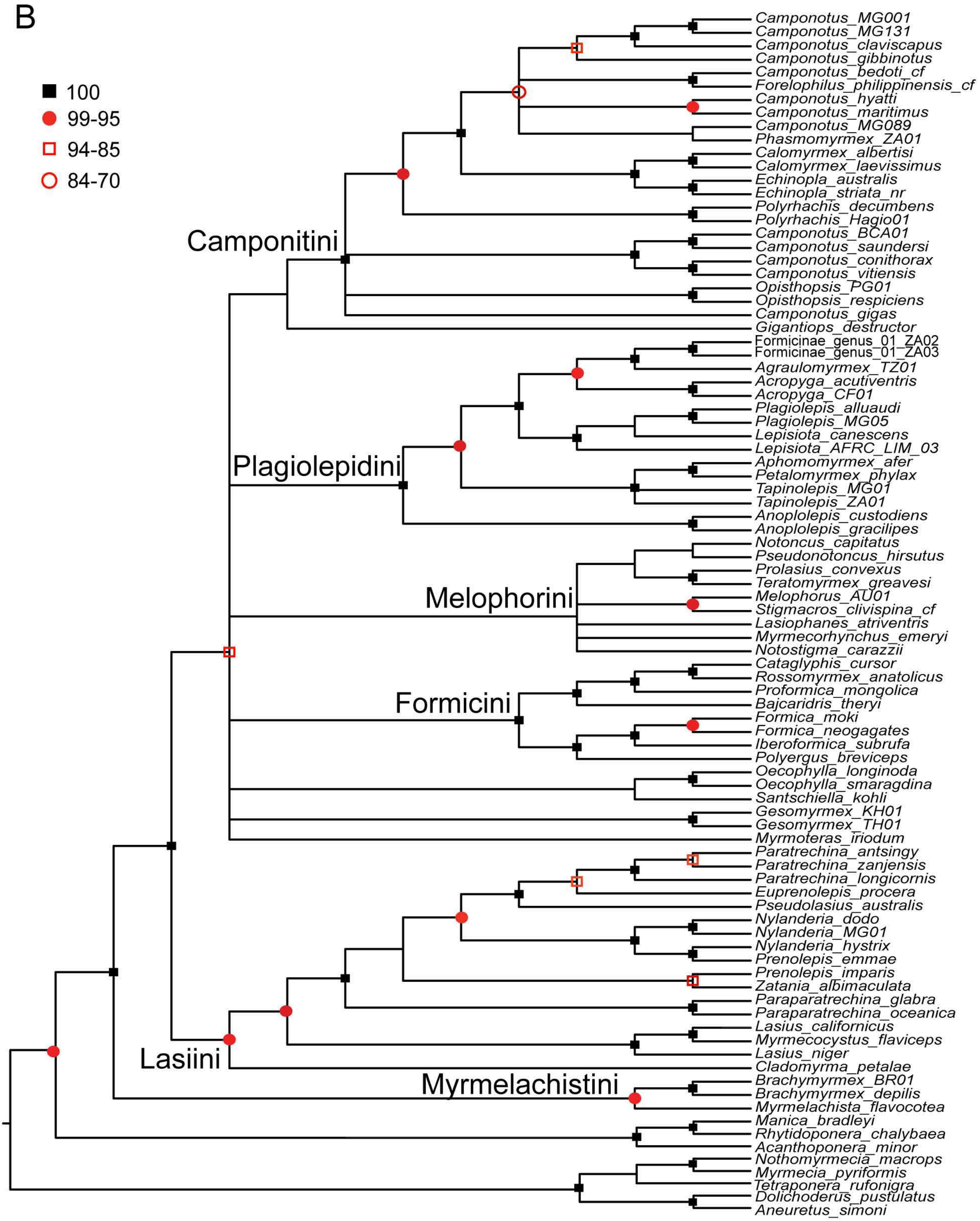
B
100
99-95
94-85
84-70
Camponitini
Plagiolepidini
Melophorini
Formicini
Lasiini
Myrmelachistini
Camponotus_MG001
Camponotus_MG131
Camponotus_claviscapus
Camponotus_gibbinotus
Camponotus_bedoti_cf
Forelophilus_philippinensis_cf
Camponotus_hyatti
Camponotus_maritimus
Camponotus_MG089
Phasmomyrmex_ZA01
Calomyrmex_albertisi
Calomyrmex_laevissimus
Echinopla_australis
Echinopla_striata_nr
Polyrhachis_decumbens
Polyrhachis_Hagio01
Camponotus_BCA01
Camponotus_saundersi
Camponotus_conithorax
Camponotus_vitiensis
Opisthopsis_PG01
Opisthopsis_respiciens
Camponotus_gigas
Gigantiops_destructor
Formicinae_genus_01_ZA02
Formicinae_genus_01_ZA03
Agraulomyrmex_TZ01
Acropyga_acutiventris
Acropyga_CF01
Plagiolepis_alluaudi
Plagiolepis_MG05
Lepisiota_canescens
Lepisiota_AFRC_LIM_03
Aphomomyrmex_afer
Petalomyrmex_phylax
Tapinolepis_MG01
Tapinolepis_ZA01
Anoplolepis_custodiens
Anoplolepis_gracilipes
Notoncus_capitatus
Pseudonotoncus_hirsutus
Prolasius_convexus
Teratomyrmex_greavesi
Melophorus_AU01
Stigmacros_clivispina_cf
Lasiophanes_atriventris
Myrmecorhynchus_emeryi
Notostigma_carazzii
Cataglyphis_cursor
Rossomyrmex_anatolicus
Proformica_mongolica
Bajcaridris_theryi
Formica_moki
Formica_neogagates
Iberoformica_subrufa
Polyergus_breviceps
Oecophylla_longinoda
Oecophylla_smaragdina
Santschiella_kohli
Gesomyrmex_KH01
Gesomyrmex_TH01
Myrmoteras_iriodum
Paratrechina_antsingy
Paratrechina_zanjensis
Paratrechina_longicornis
Euprenolepis_procera
Pseudolasius_australis
Nylanderia_dodo
Nylanderia_MG01
Nylanderia_hystrix
Prenolepis_emmae
Prenolepis_imparis
Zatania_albimaculata
Paraparatrechina_glabra
Paraparatrechina_oceanica
Lasius_californicus
Myrmecocystus_flaviceps
Lasius_niger
Cladomyrma_petalae
Brachymyrmex_BR01
Brachymyrmex_depilis
Myrmelachista_flavocotea
Manica_bradleyi
Rhytidoponera_chalybaea
Acanthoponera_minor
Nothomyrmecia_macrops
Myrmecia_pyriformis
Tetraponera_rufonigra
Dolichoderus_pustulatus
Aneuretus_simoni

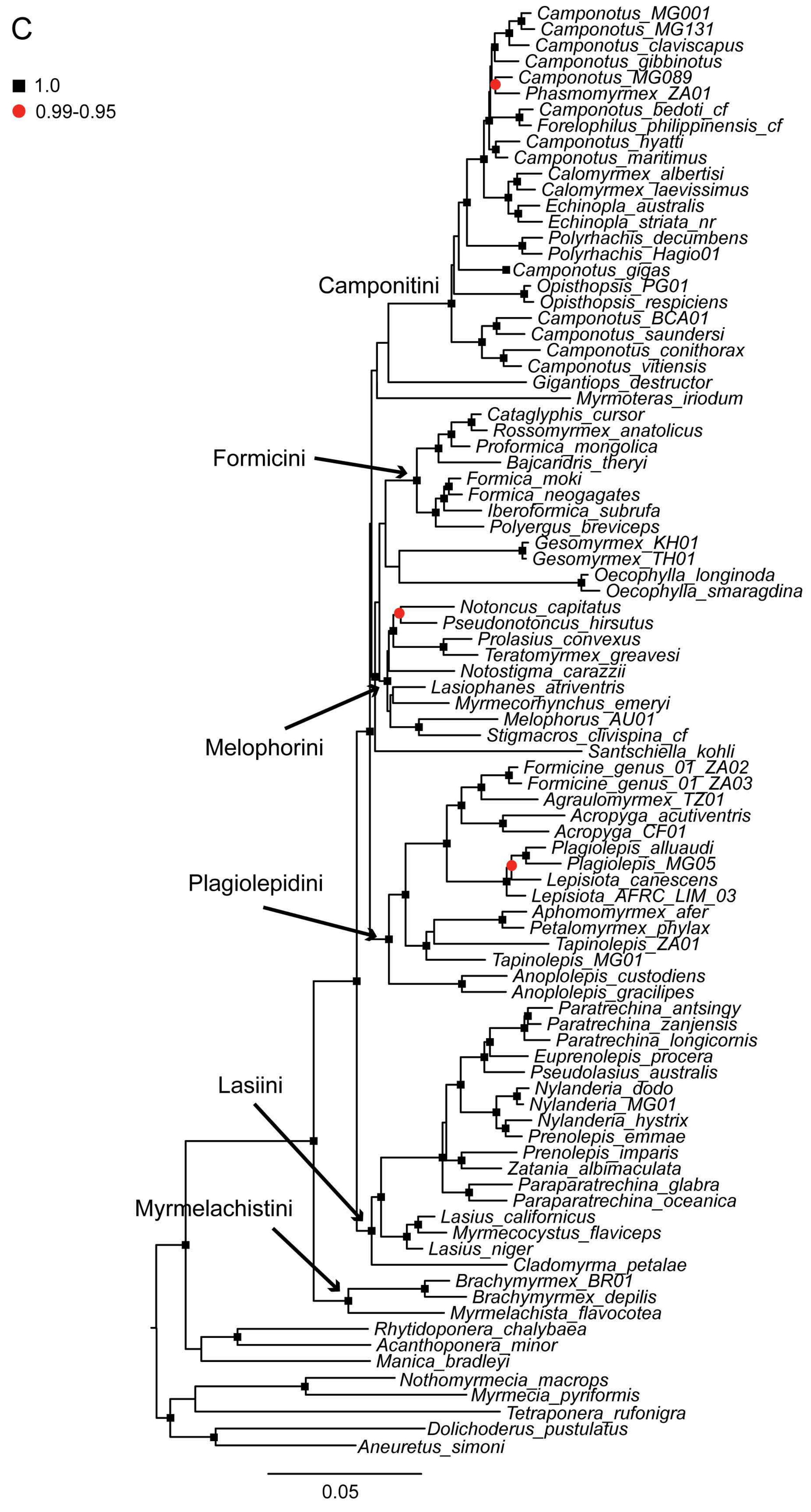
C
1.0
0.99-0.95
Camponotus_MG001
Camponotus_MG131
Camponotus_claviscapus
Camponotus_gibbinotus
Camponotus_MG089
Phasmomyrmex_ZA01
Camponotus_bedoti_cf
Forelophilus_philippinensis_cf
Camponotus_hyatti
Camponotus_maritimus
Calomyrmex_albertisi
Calomyrmex_laevissimus
Echinopla_australis
Echinopla_striata_nr
Polyrhachis_decumbens
Polyrhachis_Hagio01
Camponotus_gigas
Opisthopsis_PG01
Opisthopsis_respiciens
Camponotus_BCA01
Camponotus_saundersi
Camponotus_conithorax
Camponotus_vitiensis
Gigantiops_destructor
Myrmoteras_iriodum
Camponitini
Cataglyphis_cursor
Rossomyrmex_anatolicus
Proformica_mongolica
Bajcaridris_theryi
Formicini
Formica_moki
Formica_neogagates
Iberoformica_subrufa
Polyergus_breviceps
Gesomyrmex_KH01
Gesomyrmex_TH01
Oecophylla_longinoda
Oecophylla_smaragdina
Notoncus_capitatus
Pseudonotoncus_hirsutus
Prolasius_convexus
Teratomyrmex_greavesi
Notostigma_carazzii
Lasiophanes_atriventris
Myrmecorhynchus_emeryi
Melophorus_AU01
Stigmacros_clivispina_cf
Santschiella_kohli
Melophorini
Formicine_genus_01_ZA02
Formicine_genus_01_ZA03
Agraulomyrmex_TZ01
Acropyga_acutiventris
Acropyga_CF01
Plagiolepis_alluaudi
Plagiolepis_MG05
Lepisiota_canescens
Lepisiota_AFRC_LIM_03
Plagiolepidini
Aphomomyrmex_afer
Petalomyrmex_phylax
Tapinolepis_ZA01
Tapinolepis_MG01
Anoplolepis_custodiens
Anoplolepis_gracilipes
Paratrechina_antsingy
Paratrechina_zanjensis
Paratrechina_longicornis
Euprenolepis_procera
Pseudolasius_australis
Nylanderia_dodo
Nylanderia_MG01
Nylanderia_hystrix
Prenolepis_emmae
Prenolepis_imparis
Zatania_albimaculata
Paraparatrechina_glabra
Paraparatrechina_oceanica
Lasiini
Lasius_californicus
Myrmecocystus_flaviceps
Lasius_niger
Cladomyrma_petalae
Myrmelachistini
Brachymyrmex_BR01
Brachymyrmex_depilis
Myrmelachista_flavocotea
Rhytidoponera_chalybaea
Acanthoponera_minor
Manica_bradleyi
Nothomyrmecia_macrops
Myrmecia_pyriformis
Tetraponera_rufonigra
Dolichoderus_pustulatus
Aneuretus_simoni
0.05
