# Supplementary material for: Phylogenomic methods outperform traditional multi-locus approaches in resolving deep evolutionary history: a case study of formicine ants
Source: BMC Evol Biol. 2015 Dec 4;15:271. doi: 10.1186/s12862-015-0552-5 (PMC4670518; doi:10.1186/s12862-015-0552-5)
Supplement: Additional file 9: — Phylogenetic trees from analyses not illustrated in the main text, continued. Additional results from Maximum Likelihood analyses on UCE data subsets. (PDF 669 kb) [file 12862_2015_552_MOESM9_ESM.pdf]

Additional file 9: Phylogenetic trees from analyses not illustrated in the main text, continued. A) RAxML best tree for UCE-95% data set; B) RAxML best tree for UCE-100best data set. Both trees with bootstrap values from respective bootstrap analyses.

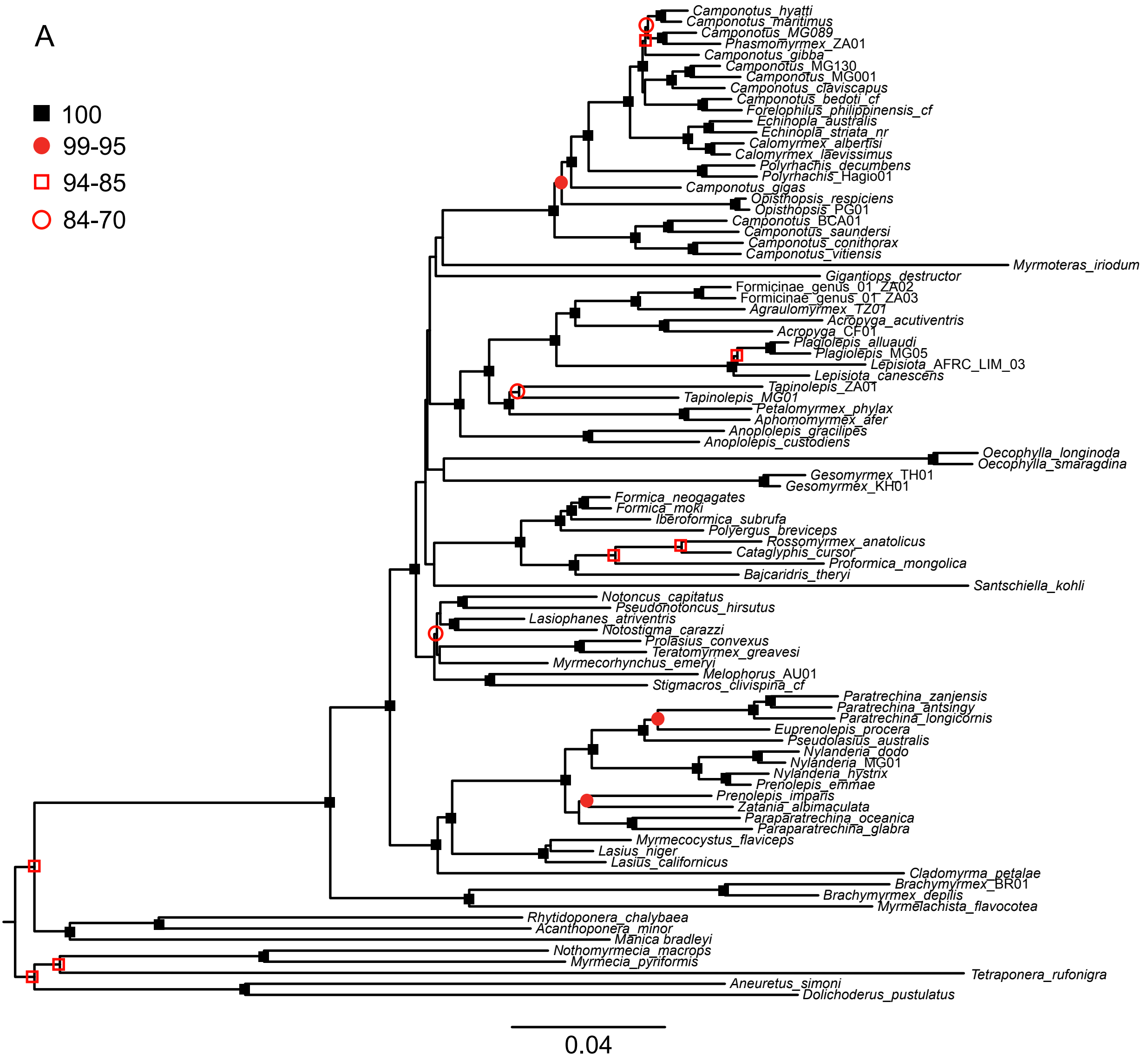

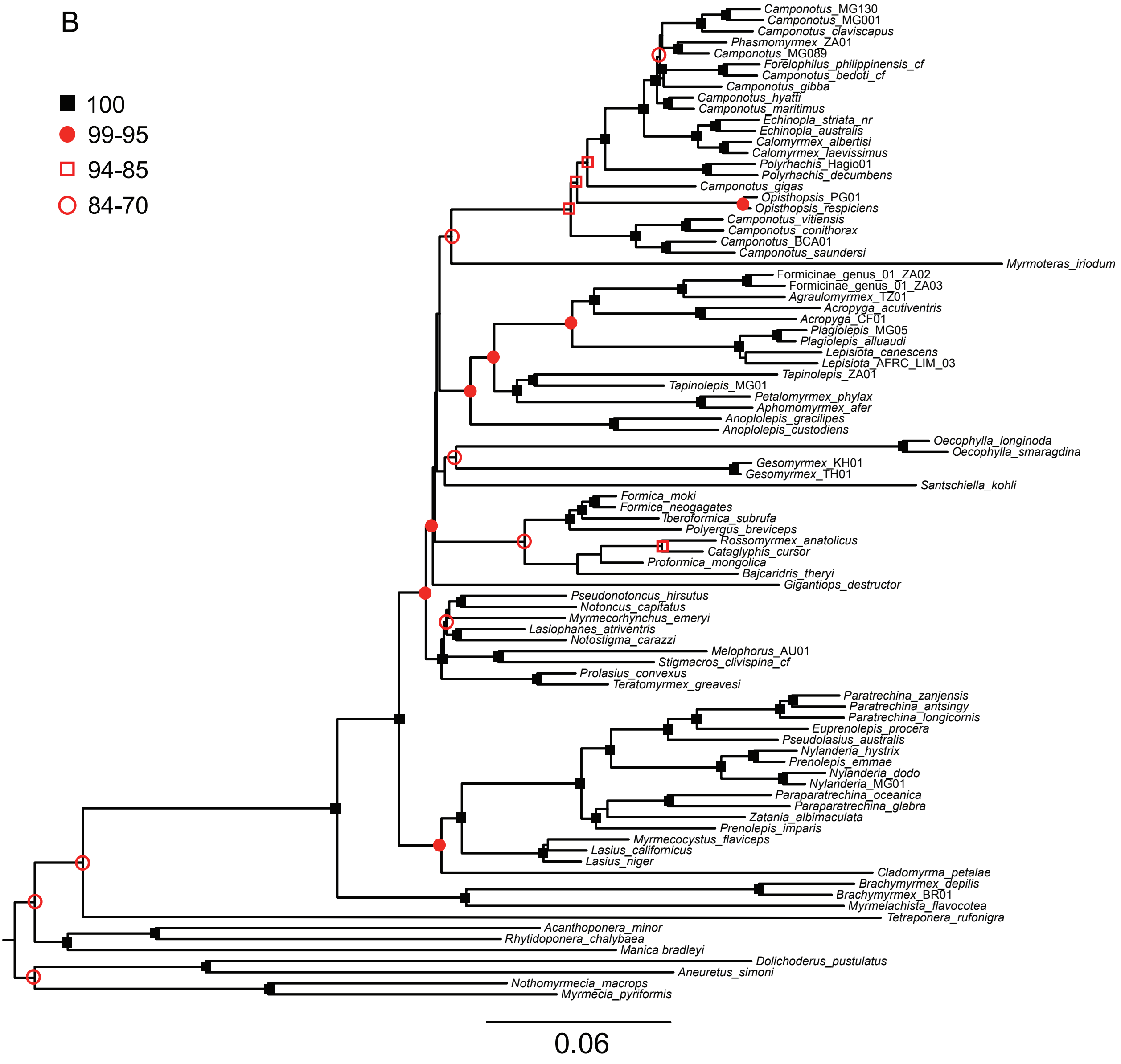
B
100
99-95
94-85
84-70
Camponotus_MG130
Camponotus_MG001
Camponotus_claviscapus
Phasmomyrmex_ZA01
Camponotus_MG089
Forelophilus_philippinensis_cf
Camponotus_bedoti_cf
Camponotus_gibba
Camponotus_hyatti
Camponotus_maritimus
Echinopla_striata_nr
Echinopla_australis
Calomyrmex_albertisi
Calomyrmex_laevissimus
Polyrhachis_Hagio01
Polyrhachis_decumbens
Camponotus_gigas
Opisthopsis_PG01
Opisthopsis_respiciens
Camponotus_vitiensis
Camponotus_conithorax
Camponotus_BCA01
Camponotus_saundersi
Myrmoteras_iriodum
Formicinae_genus_01_ZA02
Formicinae_genus_01_ZA03
Agraulomyrmex_TZ01
Acropyga_acutiventris
Acropyga_CF01
Plagiolepis_MG05
Plagiolepis_alluaudi
Lepisiota_canescens
Lepisiota_AFRC_LIM_03
Tapinolepis_ZA01
Tapinolepis_MG01
Petalomyrmex_phylax
Aphomomyrmex_afer
Anoplolepis_gracilipes
Anoplolepis_custodiens
Oecophylla_longinoda
Oecophylla_smaragdina
Gesomyrmex_KH01
Gesomyrmex_TH01
Santschiella_kohli
Formica_moki
Formica_neogagates
Iberoformica_subrufa
Polyergus_breviceps
Rossomyrmex_anatolicus
Cataglyphis_cursor
Proformica_mongolica
Bajcaridris_theryi
Gigantiops_destructor
Pseudonotoncus_hirsutus
Notoncus_capitatus
Myrmecorhynchus_emeryi
Lasiophanes_atriventris
Notostigma_carazzi
Melophorus_AU01
Stigmacros_clivispina_cf
Prolasius_convexus
Teratomyrmex_greavesi
Paratrechina_zanjensis
Paratrechina_antsingy
Paratrechina_longicornis
Euprenolepis_procera
Pseudolasius_australis
Nylanderia_hystrix
Prenolepis_emmae
Nylanderia_dodo
Nylanderia_MG01
Paraparatrechina_oceanica
Paraparatrechina_glabra
Zatania_albimaculata
Prenolepis_imparis
Myrmecocystus_flaviceps
Lasius_californicus
Lasius_niger
Cladomyrma_petalae
Brachymyrmex_depilis
Brachymyrmex_BR01
Myrmelachista_flavocotea
Tetraponera_rufonigra
Acanthoponera_minor
Rhytidoponera_chalybaea
Manica bradleyi
Dolichoderus_pustulatus
Aneuretus_simoni
Nothomyrmecia_macrops
Myrmecia_pyriformis
0.06
