# Supplementary material for: Phylogenomic methods outperform traditional multi-locus approaches in resolving deep evolutionary history: a case study of formicine ants
Source: BMC Evol Biol. 2015 Dec 4;15:271. doi: 10.1186/s12862-015-0552-5 (PMC4670518; doi:10.1186/s12862-015-0552-5)

Additional file 10: Summary of taxon exclusion experiments. Summary sketches of phylogenetic relationships contrasting the placement of the seven problematic taxa between UCE and 10-gene data set. Panels A–D UCE-70% data set, RAxML; panels E–H 10-gene data set, Bayesian. A and E: *Santschiella, Myrmoteras, Gesomyrmex* and *Oecophylla* excluded; B and F: *Santschiella* and *Myrmoteras* excluded; C and G: *Santschiella* excluded; D and H: *Myrmoteras* excluded. Black squares denote BS: 100/ PP: 1.0, red open squares BS: 94–85, red circles BS: 99–95/ PP: 0.99–0.95.

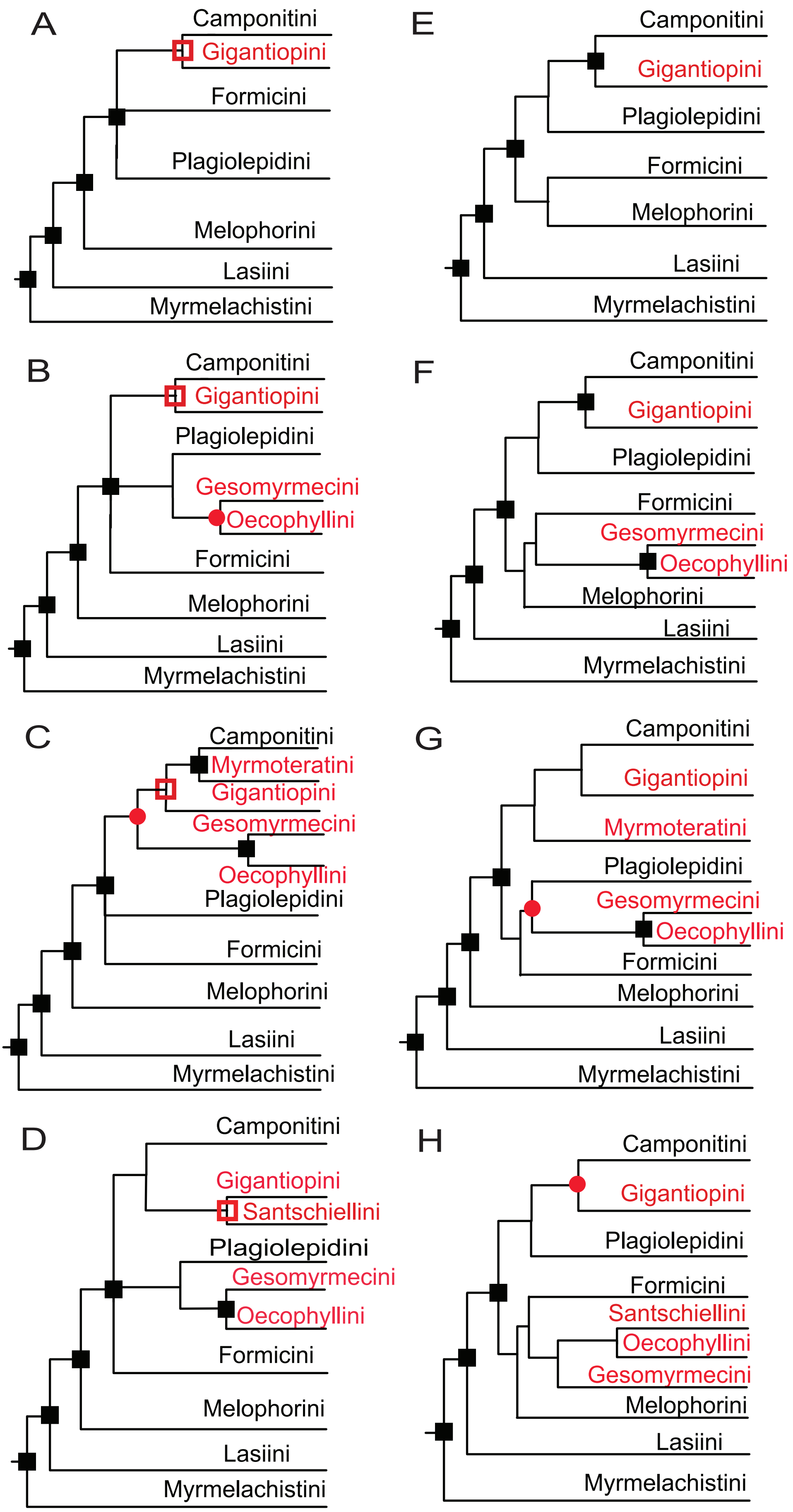

Supplement: Additional file 10: — Summary of taxon exclusion experiments. Summary sketches of phylogenetic relationships contrasting the placement of the seven rogue taxa between UCE and 10-gene data set. (PDF 248 kb) [file 12862_2015_552_MOESM10_ESM.pdf]
