# Supplementary material for: Phylogenomic methods outperform traditional multi-locus approaches in resolving deep evolutionary history: a case study of formicine ants
Source: BMC Evol Biol. 2015 Dec 4;15:271. doi: 10.1186/s12862-015-0552-5 (PMC4670518; doi:10.1186/s12862-015-0552-5)
Supplement: Additional file 11: — Comprehensive results of ancestral range estimations under the DEC and S-DEC models. Extended version of Table 2 including all crown group ancestral ranges as estimated with the DEC and S-DEC models implemented in RASP. (PDF 97 kb) [file 12862_2015_552_MOESM11_ESM.pdf]

**Additional file 11: Comprehensive results of ancestral range estimations under the DEC and S-DEC models.** Extended version of Table 2. Crown group ancestral ranges as estimated with the DEC and S-DEC models implemented in RASP. Node numbers correspond to Figure 4; only the three ranges with highest probability are shown. T = Neotropical, N = Neartic, P = Palearctic, E = Afrotropical, O = Indomalayan, A = Australasian.

| **Node** | **PP** | **Clade** | **S-DEC** | | **DEC** | |
|---|---|---|---|---|---|---|
| | | | **Area** | **Prob.** | **Area** | **Prob.** |
| 83 | 1 | *Brachymyrmex* | T | 62.62 | T | 63.02 |
| | | | TN | 37.38 | TN | 36.98 |
| 84 | 1 | Myrmelachistini | T | 95.27 | T | 100 |
| | | | TN | 4.73 | | |
| 85 | 1 | *Paraparatrechina* | EO | 42.14 | EO | 40.44 |
| | | | EOA | 34.66 | EOA | 39.21 |
| | | | EA | 19.57 | EA | 20.35 |
| 86 | 0.59 | *Paraparatrechina -* | TE | 29.83 | TE | 31.43 |
| | | *Zatania* | TEO | 22.4 | TEO | 27.55 |
| | | | E | 17.47 | TEOA | 15.47 |
| 87 | 1 | *Prenolepis* (*Zatania - Paraparatechina*) | E | 22.33 | E | 19.23 |
| | | | EO | 12.4 | EO | 13.22 |
| | | | O | 7.99 | O | 11.67 |
| 88 | 1 | *Nylanderia dodo - Nylanderia* MG01 | TEOA | 40.38 | TEOA | 38.68 |
| | | | EOA | 19.3 | EOA | 21.96 |
| | | | EO | 7.38 | EO | 9.12 |
| 89 | 1 | *Nylanderia hystrix - Prenolepis emmae* | TO | 41.16 | TO | 41.41 |
| | | | TNO | 40.09 | TNO | 39.23 |
| | | | NO | 18.56 | NO | 19.36 |
| 90 | 1 | *Nylanderia* | TEO | 22.93 | TEO | 22.28 |
| | | | O | 20.69 | O | 16.99 |
| | | | TOA | 10.81 | TOA | 11.51 |
| 91 | 1 | *Paratrechina zanjensis - Paratrechina antsingy* | E | 100 | E | 100 |
| 92 | 1 | *Paratrechina longicornis* (*Paratrechina zanjensis - Paratrechina antsingy)* | E | 100 | E | 100 |
| 93 | 1 | *Euprenolepis procera - Paratrechina* | EO | 95.63 | EO | 100 |
| | | | E | 3.18 | | |
| | | | O | 1.2 | | |
| 94 | 1 | *Pseudolasius australis* (*Euprenolepis (P. longicornis (P. zanjensis - P. antsingy*) | EO | 38.64 | O | 40.77 |

| Node | PP | Clade | S-DEC | | DEC | |
|---|---|---|---|---|---|---|
| | | | **Area** | **Prob.** | **Area** | **Prob.** |
| | | | O | 37.73 | EO | 38.98 |
| | | | E | 22.16 | E | 20.25 |
| 95 | 1 | subtending nodes 88-94 | O | 54.75 | O | 58.97 |
| | | | E | 24.17 | E | 21.91 |
| | | | EO | 18.38 | EO | 19.12 |
| 96 | 1 | subtending nodes 85-95 | EO | 19.04 | EO | 20.79 |
| | | | O | 15.28 | O | 18.51 |
| | | | PEO | 10.86 | PEO | 13.27 |
| 97 | 1 | *Lasius californicus - Myrmecocystus flaviceps* | N | 100 | N | 100 |
| 98 | 1 | *Lasius niger* (*Lasius californicus - Myrmecocystus flaviceps*) | NP | 76.52 | NP | 81.76 |
| | | | N | 23.48 | N | 18.24 |
| 99 | 1 | subtending nodes 88-98 | PO | 32.54 | PO | 43.91 |
| | | | PEO | 17.48 | PEO | 22.4 |
| | | | O | 9.12 | PE | 9.91 |
| 100 | 1 | Lasiini | O | 46.54 | O | 49.21 |
| | | | PO | 25.31 | PO | 29.4 |
| | | | PEO | 10.58 | PEO | 11.99 |
| 101 | 1 | *Lasiophanes atriventris - Notostigma carazzii* | TA | 60.91 | TA | 72.55 |
| | | | A | 39.09 | A | 27.45 |
| 102 | 1 | *Pseudonotoncus hirsutus - Notoncus capitatus* | A | 100 | A | 100 |
| 103 | 0.64 | *Myrmecorhynchus emeryi (Pseudonotoncus hirsutus - Notoncus capitatus)* | A | 100 | A | 100 |
| 104 | 1 | subtending nodes 101-103 | A | 97.85 | A | 100 |
| | | | TA | 2.15 | | |
| 105 | 1 | *Melophorus*_AU01 - *Stigmacros clivispina* | A | 100 | A | 100 |
| 106 | 0.97 | subtending nodes 101-105 | A | 99.85 | A | 100 |
| | | | TA | 0.15 | | |
| 107 | 1 | *Prolasius convexus - Teratomyrmex greavesi* | A | 100 | A | 100 |
| 108 | 1 | Melophorini | A | 99.93 | A | 100 |
| | | | TA | 0.07 | | |
| 109 | 1 | *Gesomyrmex* | O | 100 | O | 100 |
| 110 | 1 | *Oecophylla* | EOA | 75.93 | EOA | 78.06 |
| | | | EO | 23.71 | EO | 21.94 |
| | | | EA | 0.35 | | |

| Node | PP | Clade | S-DEC | | DEC | |
|---|---|---|---|---|---|---|
| | | | Area | Prob. | Area | Prob. |
| 111 | 1 | *Gesomyrmex - Oecophylla* | O | 60.05 | O | 52.12 |
| | | | EO | 29 | EO | 37.96 |
| | | | E | 9.57 | E | 9.92 |
| 112 | 0.85 | subtending nodes 109-111 | E | 35.65 | EO | 39.07 |
| | | | O | 33.42 | E | 35.39 |
| | | | EO | 30.84 | O | 25.54 |
| 113 | 1 | *Camponotus vitiensis - C. conithorax* | OA | 62.38 | OA | 63.69 |
| | | | A | 37.62 | A | 36.31 |
| 114 | 1 | *Camponotus*_BCA01 - *Camponotus saundersi* | PO | 43.92 | PO | 42.84 |
| | | | NPO | 24.71 | NPO | 25.49 |
| | | | TNPO | 24.47 | TNPO | 24.35 |
| 115 | 1 | *Colobopsis* (subtending nodes 113-114) | O | 31.45 | O | 27.67 |
| | | | OA | 18.2 | OA | 18.6 |
| | | | PO | 13.48 | PO | 13.36 |
| 116 | 1 | *Opisthopsis* | A | 100 | A | 100 |
| 117 | 1 | *Polyrhachis* | OA | 38.07 | OA | 40.84 |
| | | | EOA | 26.12 | EOA | 24.87 |
| | | | O | 18.52 | O | 15.97 |
| 118 | 1 | *Camponotus maritimus - C. hyatti* | TN | 25.05 | TN | 28.54 |
| | | | NP | 21.28 | NP | 24.98 |
| | | | TNP | 20.26 | TNP | 17.55 |
| 119 | 1 | *Phasmomyrmex ZA01 - Camponotus MG089* | E | 100 | E | 100 |
| 120 | 1 | *Camponotus MG131 - C. MG001* | E | 100 | E | 100 |
| 121 | 1 | *C. claviscapus* (*Camponotus MG131 - Camponotus MG001*) | TE | 99.85 | TE | 100 |
| | | | E | 0.15 | | |
| 122 | 1 | subtending nodes 119-124 | E | 57.54 | E | 59.19 |
| | | | TE | 42.46 | TE | 40.81 |
| 123 | 1 | *Forelophilus philippinensis_cf - Camponotus bedoti_cf* | O | 58.23 | O | 57.59 |
| | | | OA | 41.77 | OA | 42.41 |
| 124 | 1 | *Camponotus gibbonotus (Forelophilus philippinensis_cf - C. bedoti_cf)* | O | 53.74 | O | 52.11 |
| | | | OA | 28.97 | OA | 29.32 |
| | | | A | 17.29 | A | 18.57 |
| 125 | 1 | subtending nodes 118-125 | EO | 32.33 | EO | 33.28 |
| | | | TEO | 24.69 | EOA | 23.76 |

| Node | PP | Clade | S-DEC | | DEC | |
|---|---|---|---|---|---|---|
| | | | **Area** | **Prob.** | **Area** | **Prob.** |
| | | | EOA | 23.45 | TEO | 22.49 |
| 126 | 1 | *Camponotus* (as redefined) | TEO | 26.87 | PEO | 26.68 |
| | | | PEO | 25.08 | TEO | 25.73 |
| | | | PEOA | 15.42 | PEOA | 15.8 |
| 127 | 1 | *Echinopla* | A | 51.44 | A | 50.07 |
| | | | OA | 48.56 | OA | 49.93 |
| 128 | 1 | *Calomyrmex* | A | 100 | A | 100 |
| 129 | 1 | *Echinopla - Calomyrmex* | A | 69.83 | A | 68.46 |
| | | | OA | 30.17 | OA | 31.54 |
| 130 | 1 | subtending nodes 118-129 | TEOA | 16.83 | A | 13.67 |
| | | | PEOA | 13.85 | TEOA | 12.86 |
| | | | A | 13.66 | OA | 11.22 |
| 131 | 1 | subtending nodes 117-130 | O | 32.18 | OA | 34.26 |
| | | | OA | 29.68 | O | 29.87 |
| | | | A | 16.71 | A | 16.96 |
| 132 | 1 | subtending nodes 117-131 | O | 43.54 | OA | 47.96 |
| | | | OA | 40.43 | O | 40.95 |
| | | | EOA | 9.15 | EOA | 11.09 |
| 133 | 1 | subtending nodes 116-132 | OA | 47.12 | OA | 51.43 |
| | | | A | 21.81 | A | 22.99 |
| | | | O | 21.56 | O | 16.03 |
| 134 | 1 | Camponotini | O | 43.38 | O | 45.51 |
| | | | OA | 41.61 | OA | 43.86 |
| | | | EOA | 8.78 | EOA | 10.63 |
| 135 | 1 | *Myrmoteras iriodum* - Camponotini | O | 89.98 | O | 100 |
| | | | EO | 7.43 | | |
| | | | OA | 1.48 | | |
| 136 | 1 | *Anoplolepis* | EO | 99.09 | EO | 100 |
| | | | E | 0.88 | | |
| | | | O | 0.04 | | |
| 137 | 1 | *Petalomyrmex - Aphomomyrmex* | E | 100 | E | 100 |
| 138 | 1 | *Tapinolepis* | E | 100 | E | 100 |
| 139 | | subtending nodes 137-138 | E | 100 | E | 100 |
| 140 | 1 | *Plagiolepis* | PEOA | 48.72 | PEOA | 51.62 |
| | | | PEO | 13.88 | PEO | 17.62 |
| | | | E | 13.65 | EOA | 14.79 |
| 141 | 0.93 | *Lepisiota* | E | 99.67 | E | 100 |
| | | | EO | 0.2 | | |
| | | | PE | 0.11 | | |

| Node | PP | Clade | S-DEC | | DEC | |
|---|---|---|---|---|---|---|
| | | | **Area** | **Prob.** | **Area** | **Prob.** |
| 142 | 1 | *Plagiolepis - Lepisiota* | E | 96.94 | E | 100 |
| | | | EO | 3.06 | | |
| 143 | 1 | Formicinae_genus_01 | E | 100 | E | 100 |
| 144 | 1 | *Agraulomyrmex* - Formicinae_genus_01 | E | 100 | E | 100 |
| 145 | 1 | *Acropyga* | E | 31.59 | E | 17.32 |
| | | | TNPEOA | 11.15 | TPEOA | 6.25 |
| | | | TPEOA | 6.93 | TNPEOA | 6.07 |
| 146 | 1 | subtending nodes 143-145 | E | 99.5 | E | 100 |
| | | | TE | 0.17 | | |
| | | | EO | 0.16 | | |
| 147 | 1 | subtending nodes 141-146 | E | 99.41 | E | 100 |
| | | | EO | 0.59 | | |
| 148 | 1 | subtending nodes 137-147 | E | 99.17 | E | 100 |
| | | | EO | 0.83 | | |
| 149 | 1 | Plagiolepidini | EO | 52.91 | E | 51.22 |
| | | | E | 45.65 | EO | 48.78 |
| | | | O | 1.44 | | |
| 150 | 0.98 | subtending nodes 113-149 | O | 48.67 | EO | 44.99 |
| | | | EO | 35.3 | O | 42.25 |
| | | | E | 14.63 | E | 12.76 |
| 151 | 0.82 | subtending nodes 109-149 | EO | 43.67 | EO | 58 |
| | | | O | 32.67 | O | 20.48 |
| | | | E | 19.45 | E | 13.07 |
| 152 | 1 | *Formica* | NP | 68.21 | NP | 71.78 |
| | | | TNP | 18.48 | TNP | 17.7 |
| | | | N | 12.92 | N | 10.52 |
| 153 | 1 | *Iberoformica - Formica* | P | 74.13 | P | 78.95 |
| | | | NP | 25.69 | NP | 21.05 |
| | | | N | 0.18 | | |
| 154 | 0.99 | *Polyergus* (*Formica - Iberoformica*) | P | 77.17 | P | 80.89 |
| | | | NP | 22.73 | NP | 19.11 |
| | | | N | 0.09 | | |
| 155 | 0.99 | *Rossomyrmex - Cataglyphis* | P | 53.05 | P | 40.48 |
| | | | PEO | 20.73 | PEO | 26.92 |
| | | | PO | 13.12 | PO | 16.49 |
| 156 | 0.5 | *Bajcaridris - Proformica* | P | 100 | P | 100 |
| 157 | 0.97 | *(Rossomyrmex - Cataglyphis) (Bajcaridris - Proformica)* | P | 95.13 | P | 100 |
| | | | PO | 2.03 | | |

| Node | PP | Clade | S-DEC | | DEC | |
|---|---|---|---|---|---|---|
| | | | Area | Prob. | Area | Prob. |
| | | | PE | 1.7 | | |
| 158 | 0.58 | Formicini | P | 89.94 | P | 100 |
| | | | NP | 7.13 | | |
| | | | PO | 1.54 | | |
| 159 | 0.58 | subtending nodes 109-158 | PEO | 63.08 | PEO | 63.25 |
| | | | PO | 15.19 | PO | 15.77 |
| | | | PEOA | 10.12 | PEOA | 10.74 |
| 160 | 1 | subtending nodes 109-159 | TPEO | 40.37 | TPEO | 61.58 |
| | | | TPO | 24.11 | TPO | 15.54 |
| | | | TPE | 14.17 | TPEOA | 12.11 |
| 161 | 1 | subtending nodes 101-160 | TPEOA | 43.01 | TPEOA | 67.84 |
| | | | TPOA | 25.28 | TPOA | 18.25 |
| | | | TPEA | 15.58 | TPEA | 13.92 |
| 162 | 1 | subtending nodes 85-161 | TPEOA | 43.57 | TPEOA | 65.41 |
| | | | TPOA | 21.67 | TPOA | 22.1 |
| | | | PEOA | 7.38 | TEOA | 12.49 |
| 163 | 0.9 | Formicinae | TPOA | 19.03 | TPEOA | 21.95 |
| | | | TPEOA | 18.26 | TPOA | 18.61 |
| | | | TEOA | 8.08 | TEOA | 9.87 |
